# Vangl2–environment interaction causes severe neural tube defects, without abnormal neuroepithelial convergent extension

**DOI:** 10.1242/dmm.049194

**Published:** 2022-01-26

**Authors:** Oleksandr Nychyk, Gabriel L. Galea, Matteo Molè, Dawn Savery, Nicholas D. E. Greene, Philip Stanier, Andrew J. Copp

**Affiliations:** 1 Developmental Biology and Cancer Research Department, UCL Great Ormond Street Institute of Child Health, London WC1N 1EH, UK; 2 Genetics and Genomic Medicine Research Department, UCL Great Ormond Street Institute of Child Health, London WC1N 1EH, UK

**Keywords:** Embryo culture, Glycosaminoglycans, Mouse, Neurulation, Planar cell polarity, Proteoglycans

## Abstract

Planar cell polarity (PCP) signalling is vital for initiation of mouse neurulation, with diminished convergent extension (CE) cell movements leading to craniorachischisis, a severe neural tube defect (NTD). Some humans with NTDs also have PCP gene mutations but these are heterozygous, not homozygous as in mice. Other genetic or environmental factors may interact with partial loss of PCP function in human NTDs. We found that reduced sulfation of glycosaminoglycans interacts with heterozygosity for the *Lp* allele of *Vangl2* (a core PCP gene), to cause craniorachischisis in cultured mouse embryos, with rescue by exogenous sulphate. We hypothesized that this glycosaminoglycan–PCP interaction may regulate CE, but, surprisingly, DiO labelling of the embryonic node demonstrates no abnormality of midline axial extension in sulfation-depleted *Lp/+* embryos. Positive-control *Lp/Lp* embryos show severe CE defects. Abnormalities were detected in the size and shape of somites that flank the closing neural tube in sulfation-depleted *Lp/+* embryos. We conclude that failure of closure initiation can arise by a mechanism other than faulty neuroepithelial CE, with possible involvement of matrix-mediated somite expansion, adjacent to the closing neural tube.

## Abstract (precis)

**Summary:** A gene–environment interaction involving the *Vangl2* gene and sulfated glycosaminoglycans prevents mouse neural tube closure. Convergent extension shaping of the embryo is unaffected, implicating other mechanism(s) in this process.

## INTRODUCTION

Neurulation is the series of embryonic events that gives rise to the closed neural tube (NT), the precursor of the brain and spinal cord. Failure of NT closure at any level of the body axis leads to neural tube defects (NTDs), in which the neural plate remains open and subsequently degenerates, resulting in loss of neural function below that body level (Copp et al., 2015). Such defects are prominent causes of perinatal mortality and postnatal disability in humans, with NTDs affecting 1 per 1000 pregnancies on average worldwide, and with much higher frequencies in some geographical locations (Zaganjor et al., 2016). In the mouse embryo, NT closure initiates (termed Closure 1) at the hindbrain/cervical boundary at the 6- to 7-somite stage, with the open neural groove closing *de novo* at the level of the 3rd somite (Sakai, 1989). Initiation of NT closure occurs at a similar stage and somite level in human embryos (O'Rahilly and Müller, 2002). Failure of Closure 1 generates the most severe type of NTD, craniorachischisis (CRN), in which the neural tube remains open from midbrain to low spine (Copp et al., 1994), a defect that comprises ∼10% of human NTDs, with a larger proportion in areas of high overall NTD prevalence (Moore et al., 1997).

Initiation of NT closure requires signalling via the planar cell polarity (PCP) pathway, a non-canonical Wnt–Dishevelled cascade, which regulates convergent extension (CE). In this process, cells intercalate in the plane of the neural plate or other epithelium, driving mediolateral narrowing and rostrocaudal elongation, which serves to shape the embryo during and following gastrulation (Skoglund and Keller, 2010). NT closure is disrupted in mice homozygous for mutations in the PCP pathway, making this the primary signalling cascade known to be required for initiation of NT closure (Wallingford, 2012). VANGL planar cell polarity 2 (*Vangl2*) is the best-understood core PCP gene, and homozygosity for the dominant negative loop-tail (*Lp*) allele (Strong and Hollander, 1949), or for a null allele (Yin et al., 2012; Ramsbottom et al., 2014), produces CRN. In contrast, single heterozygotes are usually normal, except for a looped tail and occasional spina bifida.

Double heterozygosity of *Lp* with other PCP gene mutations generally produces CRN (Murdoch et al., 2014), whereas interaction with genes outside the PCP pathway generates a range of NTDs, including exencephaly and spina bifida. For example, *Lp*/grainyhead-like transcription factor 3 (*Grhl3*) doubly heterozygous mutants develop severe spina bifida (Stiefel et al., 2007; Caddy et al., 2010; De Castro et al., 2018), while *Lp*/cordon-bleu WH2 repeat (*Cobl*) mutants exhibit exencephaly (Carroll et al., 2003). Interaction is also seen with non-genetic factors; for example, Closure 1 failure occurs at lower concentrations of the Rho kinase inhibitor Y27632 in *Lp/+* embryos than in wild-type littermates (Ybot-Gonzalez et al., 2007). Hence, the *Lp* mutation is a proven ‘sensitized detector’ that can identify both gene–gene and gene–environment interactions that lead to NTDs.

Glycosaminoglycans (GAGs) are linear, unbranched polyanionic molecules of repeating disaccharides that are covalently linked to core proteins to form proteoglycans. GAGs bind and regulate the activity of many secreted factors during development, and these interactions depend on the degree of sulfation, epimerization and acetylation of the chains (Gorsi and Stringer, 2007; Iozzo and Schaefer, 2015). The expression of GAGs is dynamically regulated throughout development (Caterson, 2012), and several studies have shown that the main sulfated GAGs synthesized during primary neurulation in the mammalian embryo are heparan sulfate (HS) and chondroitin sulfate (CS), while the main non-sulfated GAG is hyaluronan (HA) (Solursh and Morriss, 1977; Copp and Bernfield, 1988b; Yip et al., 2002). Enzymatic removal of sulfate groups from either HS or CS leads to faulty cranial neural tube closure in cultured rat embryos (Morriss-Kay and Tuckett, 1989; Tuckett and Morriss-Kay, 1989), while digestion of HA retards rat cranial closure (Morriss-Kay et al., 1986). Pre-spina bifida curly tail (*ct*) mutant mouse embryos exhibit abnormal HA accumulation (Copp and Bernfield, 1988a). These studies reveal a role for GAGs in regulating neurulation, with support from the finding that the *Lp* mutation interacts genetically with a null mutation in a HS proteoglycan, syndecan-4 (*Sdc4*), to disrupt spinal closure (Escobedo et al., 2013).

Previously, spinal NT closure was found to be affected when wild-type embryos were treated in culture with chlorate, a competitive inhibitor of GAG sulfation (Yip et al., 2002). Moreover, a preliminary study suggested that Closure 1 is also sensitive to chlorate, specifically in *Lp/+* embryos (Escobedo et al., 2013). The current study was therefore designed to investigate in detail the interaction between Vangl2 and GAG chains during initiation of NT closure, and to determine the underlying developmental mechanisms. We find that inhibition of GAG sulfation, or enzymatic removal of sulfated GAG chains, interacts with partial loss of PCP signalling to disrupt Closure 1. Strikingly, however, this effect is not mediated by faulty neuroepithelial CE, which has been found to underlie other instances of CRN in the mouse embryo (Greene et al., 1998; Ybot-Gonzalez et al., 2007; Williams et al., 2014). Hence, additional developmental mechanisms must also be critical for initiation of neural tube closure, and these may be implicated alongside CE in the pathogenesis of severe human NTDs.

## RESULTS

**Fig. 1.**
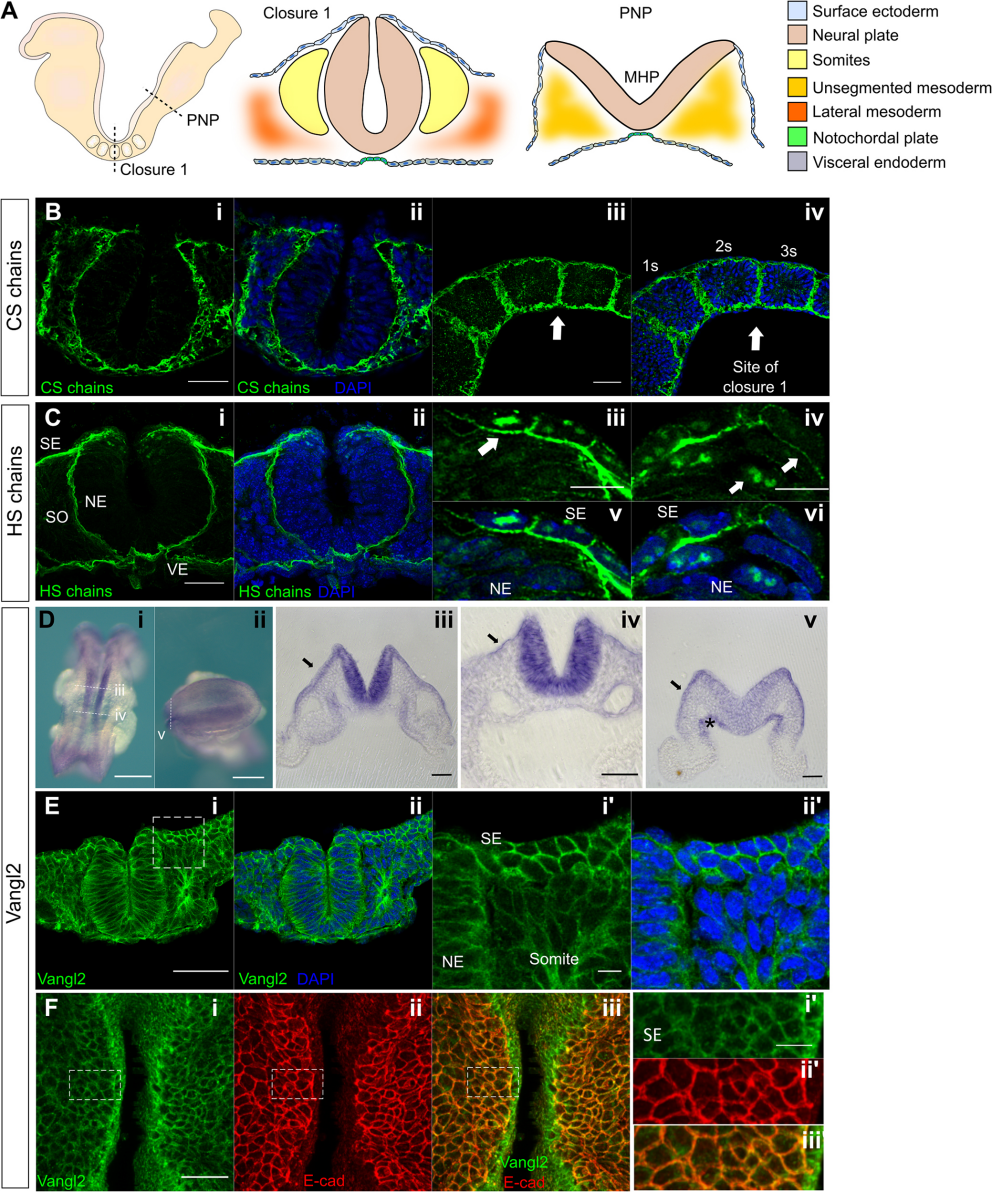
**Expression of chondroitin sulfate (CS), heparan sulfate (HS) and Vangl2 at the Closure 1 site of wild-type embryos.** (A) Schematic representation of an E8.5 mouse embryo (left), showing transverse sections through the Closure 1 region (middle) and posterior neuropore (PNP; right). A median hinge point (MHP) is prominent in the PNP, with non-bending neural plate lateral to it, whereas this is not seen at the Closure 1 site. (B,C) Immunofluorescence staining of CS (B) and HS (C) in transverse (B-i,ii,C-i,ii) and parasagittal (B-iii,iv,C-iii,iv) sections at the Closure 1 site. Both GAG chains localize to the basement membrane of NE and SE (B-i,ii,C-i,ii) (6-somite stage). CS chains are expressed at somite borders (B-iii,iv), with strong staining of HS chains at lateral junctions of SE cells (arrow in C-iii) and weak staining in NE cell membranes (arrows in C-iv). HS chains show nuclear localization in the dorsal NE and SE at the site of fusion (arrows in C-v,vi). (D) *Vangl2* mRNA expression visualized by whole-mount *in situ* hybridization (D-i,ii) with transverse vibratome sections (D-iii-v). Transcripts are present throughout the NE, especially at the site of Closure 1 (D-i,iii,iv), and less intensely at the level of the future posterior neuropore (D-ii,v) (6-somite stage). D-ii shows a dorsal view of an open PNP, anterior to left. *Vangl2* mRNA is also detected in SE (arrows in D-iii-v) and less intensely in visceral endoderm (asterisk in D-v) and paraxial mesoderm, at all levels shown. (E) Vangl2 protein is broadly expressed in NE, SE, somitic mesoderm and endoderm (6-somite stage). (F) Dorsal view of whole-mount embryo (5-somite stage) double immunostained for Vangl2 and E-cadherin (F-i,ii,iii), confirming the presence of Vangl2 in SE (F-i′,ii′,iii′). The SE and apical surface of neural plate was ‘isolated’ virtually using in-house macros. Images acquired by laser-scanning confocal microscopy using oil immersion, deconvoluted post-acquisition, and processed by single *z*-plane in C-iii,vi and F-i′,ii′,iii′. NE, neuroepithelium; SE, surface ectoderm; SO, somite; VE, visceral endoderm. ‘1s’, ‘2s’ and ‘3s’ indicate first, second and third somites, respectively. Scale bars: 10 µm (C-iii,vi,E-i′,ii′); 20 µm (F-i′,ii′iii′); 50 µm (B,C-i,ii,D-iii-v,E-i,ii); 100 µm (F-i,ii); 250 µm (D-i,ii).

The morphology of Closure 1, as visualized in transverse sections, differs from closure at other body levels (Fig. 1A). During cranial and spinal neurulation, the closing neural plate bends focally at either the midline [median hinge point (MHP); Fig. 1A] or at dorsolateral hinge points (DLHPs), or both (Morriss-Kay, 1981; Shum and Copp, 1996). In contrast, the Closure 1 site does not show focal bending at MHP or DLHPs; instead, the neural plate displays a ‘horseshoe’ morphology in cross section (Fig. 1A,B-i,ii,C-i,ii,E-i,ii). A further difference is that the closing neural folds are directly flanked by somites that have already undergone a mesenchymal–epithelial transition (Nakaya et al., 2004), and so possess an outer epithelial border (Fig. 1A,E). This contrasts with the unsegmented cranial or presomitic mesoderm that flanks the closing neural tube at other body levels. Hence, the unique morphology and neural plate–mesoderm relationship of the closure initiation site suggests that developmental mechanisms may differ between Closure 1 and other cranial and spinal levels.

### Expression of CS and HS chains, and Vangl2 during initiation of neural tube closure

Immunofluorescence analysis of sections of wild-type embryos reveals the distribution of CS and HS chains at the stage and location of Closure 1. We used the CS-56 monoclonal antibody to recognize CS, and the 10E4 monoclonal antibody for HS (Avnur and Geiger, 1984; David et al., 1992). These are specific for their respective GAG types, but do not detect the associated proteoglycan protein backbones. Both CS and HS are detected in basement membranes underlying the neuroepithelium (NE), surface ectoderm (SE) and visceral endoderm (Fig. 1B,C). Sagittal sections through the Closure 1 site show strong staining of CS chains at the somite borders and weak staining within the somitic mesoderm (Fig. 1B-iii,iv). HS staining is observed at the basolateral junctions of SE cells, and less-intense staining is present around NE cells (Fig. 1C-iii,iv). Nuclear HS staining is observed in the most dorsal NE and SE cells and in some mesodermal cells (Fig. 1C-v,vi). Double immunofluorescence analysis confirmed that both CS and HS chains colocalize with laminin in the SE and NE basement membranes (Fig. S1A,B).

Strong expression of *Vangl2* mRNA is observed at the Closure 1 site (Fig. 1D) (Doudney et al., 2005; Ybot-Gonzalez et al., 2007), with particularly intense signal in NE and lower-intensity expression in somitic, presomitic and lateral plate mesoderm. Transcripts are also detected in SE and visceral endoderm (Fig. 1D-iii-v). Immunohistochemistry confirms the widespread expression of Vangl2 protein in cell membranes of all embryonic tissues at the Closure 1 site (Fig. 1E). Double whole-mount immunofluorescence with anti-Vangl2 and anti-E-cadherin, an SE marker, verified the presence of Vangl2 in SE during neural tube closure (Fig. S2A), and confirmed the absence of Vangl2 membrane staining in *Vangl2^Lp/Lp^* (hereafter, *Lp/Lp*) mutant embryos (Fig. S2B). The expression pattern of CS and HS chains is similar between wild-type and *Lp/Lp* embryos at the Closure 1 site (Fig. 1B,C versus Fig. S1C,D). Taken together, this analysis reveals that CS and HS chains are co-expressed with Vangl2 in the NE, somitic mesoderm and SE of the Closure 1 region.

### Inhibition of GAG sulfation disrupts Closure 1 in *Vangl2^Lp/+^* and *Vangl2^flox/−^* embryos

**Fig. 2.**
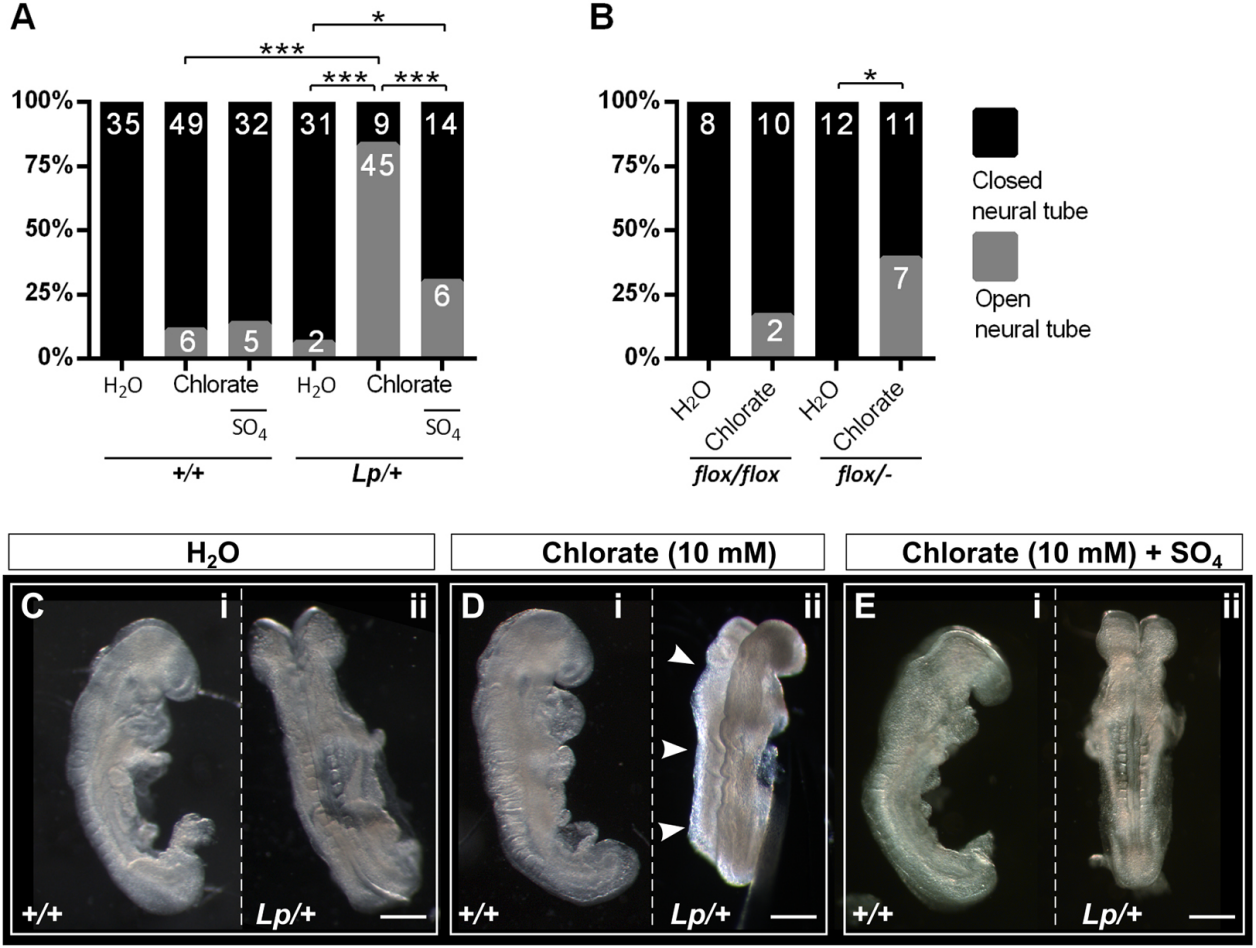
**Chlorate induces failure of Closure 1 in *Vangl2^Lp/+^* and *Vangl2^flox/−^* embryos.** (A) Experimental litters were generated by *Vangl2^Lp/+^*×*Vangl2^+/+^* matings within the CBA/Ca-derived genetic background. Percentage of open NT (grey bar sectors) versus closed NT (black bar sectors) at the Closure 1 site was determined in cultures treated with water (control groups), 10 mM chlorate (treatment groups) or 10 mM chlorate plus sodium sulfate (rescue groups). Embryo numbers are shown on bars. Treatment with chlorate prevents Closure 1 in a significantly greater proportion of *Lp/+* than *+/+* embryos (*P*<0.001). Closure 1 is successful in 100% of water-treated controls. Co-administration of sodium sulfate (SO_4_) significantly reduces the Closure 1 failure seen in *Lp/+* embryos treated with 10 mM chlorate (*P*<0.001), but not in *+/+* embryos (*P*>0.05). (B) Chlorate significantly inhibits Closure 1 in *Vangl2^flox/−^*embryos, compared with *Vangl2^flox/flox^*, although the penetrance of Closure 1 failure after 10 mM chlorate is significantly lower in *Vangl2^flox/−^* embryos (7/18) than in *Vangl2^Lp/+^* embryos (A; 45/54; *P*<0.001). (C-E) Typical embryonic morphology after 24 h culture. *Vangl2^+/+^* embryos show completion of Closure 1 in all treatment groups (C-i,D-i,E-i). *Vangl2^Lp/+^* water-treated (C-ii) and chlorate+sulfate-treated embryos (E-ii) also show Closure 1, whereas a chlorate-treated *Vangl2^Lp/+^* embryo shows entirely open neural tube (arrowheads in D-ii). *P<0.05; ***P<0.001 (Chi-square test). Scale bars: 0.5 mm.

Chlorate is a competitive, reversible inhibitor of GAG chain sulfation, commonly used to investigate the role of sulfate groups in proteoglycan function (Conrad, 2001). It has been added to cell and embryo cultures at concentrations up to 30 mM, without apparent adverse effects on GAG or protein synthesis, or cell viability (Greve et al., 1988; Humphries and Silbert, 1988; Yip et al., 2002). We performed a dose–response study in whole-embryo culture, to identify a minimum concentration of chlorate that would affect Closure 1 in *Vangl2^+/+^* and *Vangl2^Lp/+^* (hereafter, *+/+* and *Lp/+*, respectively) embryos without toxic effects on embryonic growth and development (Table S1). Concentrations of chlorate up to 10 mM had no significant adverse effects on embryonic health parameters, although 20 mM produced a significant reduction in yolk sac circulation score (Table S1). We therefore focused on 10 mM chlorate, which inhibited Closure 1 (entirely open NT) in 83.3% (45/54) of treated *Lp/+* embryos (Fig. 2A). In contrast, 6.1% (2/33) of *Lp/+* embryos treated with water only (vehicle) showed an open NT (*P*<0.0001). The same concentration of chlorate caused *+/+* embryos to develop NT closure defects in 10.9% (6/55) of cases, whereas there were no Closure 1 defects (0/35) in water-treated *+/+* embryos. Hence, 10 mM chlorate was selected for use in all subsequent culture experiments with *+/+* and *Lp/+* embryos.

A rescue experiment was performed in which embryos received 10 mM sodium sulfate in addition to 10 mM chlorate in the culture medium. Exogenous sulfate is known to restore GAG sulfation in a dose-dependent manner, without competitively inhibiting chlorate (Humphries and Silbert, 1988). The frequency of chlorate-induced Closure 1 failure in *Lp/+* embryos decreased significantly from 83.3% (45/54) in the absence of sodium sulfate to 30% (6/20) in its presence (*P*<0.0001; Fig. 2A), without any adverse effects on embryo morphology (Fig. 2C-E). Interestingly, however, the frequency of open NT in *Lp/+* embryos remained significantly higher in the chlorate+sulfate group than in the water-treated group (*P*=0.042). Hence, exogenous sulfate can rescue around half of *Lp/+* embryos from chlorate-induced Closure 1 failure, demonstrating a specific role for GAG sulfation, in combination with partial disruption of Vangl2 function.

The effect of chlorate was tested independently in a targeted transgenic line: *Vangl2^flox/−^*. Global loss of Vangl2 using this knockout allele recapitulates the *Lp/Lp* phenotype (Ramsbottom et al., 2014). *Vangl2^flox/−^* embryos exposed in culture to 10 mM chlorate failed in Closure 1 in 38.9% (7/18) of cases, whereas water-treated *Vangl2^flox/−^* embryos showed no closure defects (0/12; *P*=0.024; Fig. 2B). Strikingly, however, the penetrance of Closure 1 defects in chlorate-treated *Vangl2^flox/−^* embryos (Fig. 2B) was significantly lower (*P*<0.001) than in chlorate-treated *Lp/+* embryos (Fig. 2A). This is consistent with previous findings that *Lp* has more severe (likely dominant-negative) effects on PCP signalling compared with *Vangl2*-null alleles (Yin et al., 2012).

It is interesting that 10 mM chlorate produced a low frequency of Closure 1 failure in wild-type embryos, both in *Lp* experiments (6/55, 10.9%; Fig. 2A) and *Vangl2^flox^* experiments (2/12, 16.7%; Fig. 2B). In contrast, water-treated (control) wild-type embryos showed no closure defects in either experiment (0/35 in Fig. 2A; 0/8 in Fig. 2B). These individual experiment differences are not statistically significant (*P>*0.05), whereas the pooled wild-type data reach significance (0/43 control, 8/67 chlorate treated; *P*=0.048). Addition of exogenous sulfate did not rescue chlorate-induced NT closure defects in *+/+* embryos, in contrast to the ∼50% reduction in Closure 1 failure seen in *Lp/+* embryos (Fig. 2A). This argues against a specific requirement for GAG sulfation in Closure 1. Alternatively, chlorate could exert a general effect on embryonic health. Indeed, yolk sac circulation score was lower in the 10 mM chlorate group than in water-treated controls (Table S1), although this difference did not reach statistical significance (*P*=0.057). Hence, we are currently unable to account for the low-frequency Closure 1 failure in chlorate-treated wild-type embryos.

We asked whether a developmental ‘window’ exists when Closure 1 is susceptible to chlorate treatment. Somite number at the end of culture did not differ between genotype or treatment groups (Fig. S3A). Somite stage at the start of culture was calculated by subtracting the expected number of new somites formed during the culture period (assuming one new somite every 2 h) from the somite number at the end of culture (Fig. S3B). The great majority of *+/+* embryos completed NT closure, even when treated with chlorate. In contrast, most *Lp/+* embryos failed in NT closure after exposure to 10 mM chlorate, and this comprised 92% (22/24) of those treated from the 0- to 1-somite stage, 83% (10/12) from the 2- to 3-somite stage and 60% (3/5) from the 4- to 5-somite stage (*P*>0.05; Fig. S3C). Hence, chlorate can inhibit Closure 1 even if added only a few hours before the event.

**Fig. 3.**
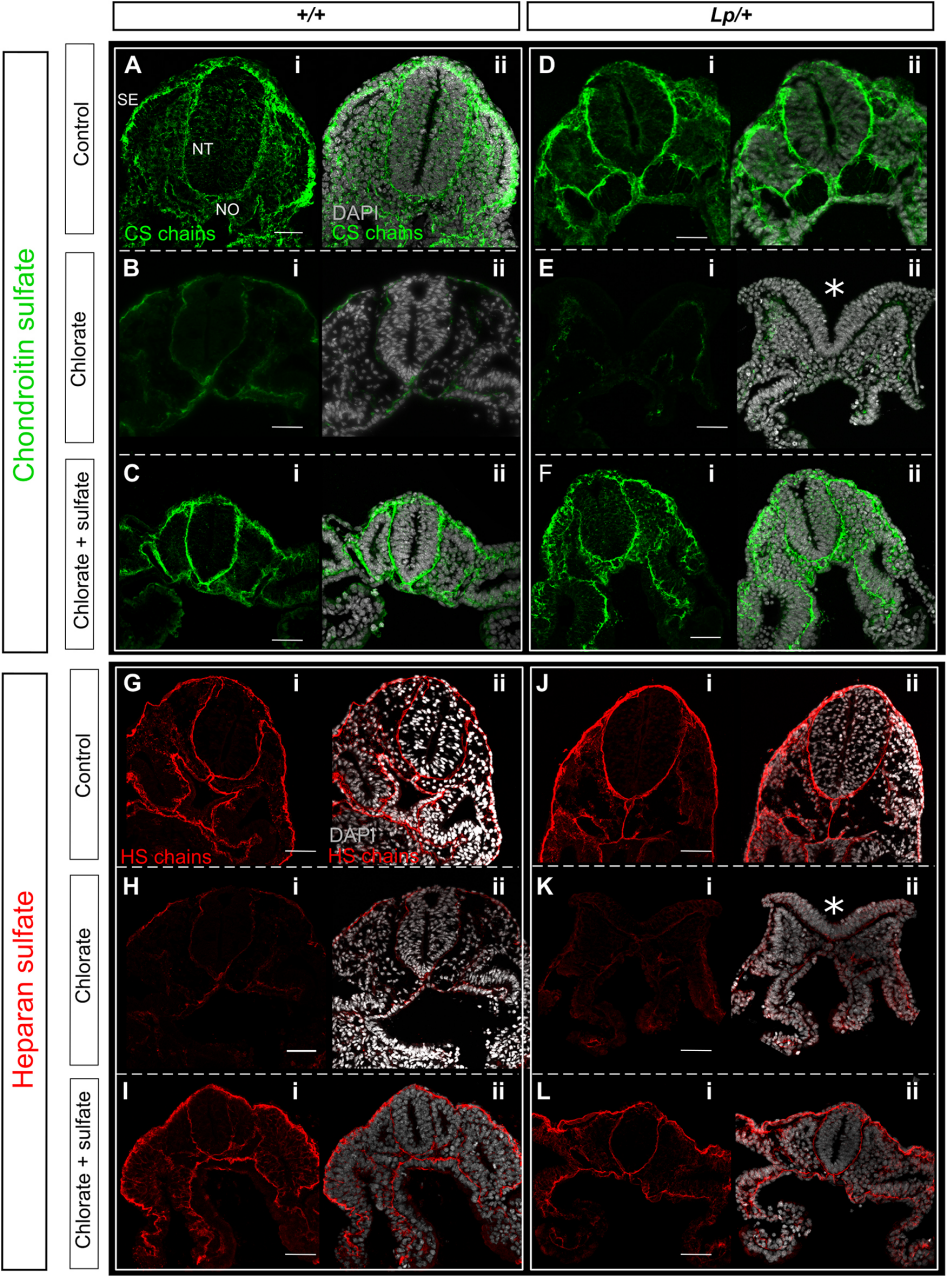
**Chlorate reduces sulfation of CS and HS chains in cultured embryos.** (A-L) Immunofluorescence analysis of CS (A-F) and HS (G-L) chains in embryos cultured in the presence of water (control), 10 mM chlorate, or 10 mM chlorate plus sodium sulfate. (A,D,G,J) Sections from control *+/+* and *Lp/+* embryos have normal distribution of CS and HS chains, which are localized to the BM underlying SE and surrounding the NT and notochord (NO); CS staining is also detected around mesenchymal cells. (B,E,H,K) Chlorate treatment dramatically reduces the staining of CS and HS chains in both genotypes and leads to failure of Closure 1 in *Lp/+* embryos (asterisks in E-ii and K-ii: open NT). (C,F,I,L) Staining of CS and HS chains in *+/+* and *Lp/+* embryos from the rescue group (chlorate plus sulfate) appears similar to that in the control group. Note the closed NT in *Lp/+* embryos. Stages shown: 12-14 somites. Scale bars: 50 µm.

Immunofluorescence analysis was used to determine the effect of chlorate on distribution of sulfated GAGs in the cultured embryos. Wild-type and *Lp/+* embryos cultured under control conditions exhibit strong CS and HS staining (Fig. 3A,D,G,J), similar to the immunostaining of wild-type embryos prior to Closure 1 (Fig. 1B,C). In contrast, CS and HS are dramatically reduced in chlorate-treated *+/+* and *Lp/+* embryos (Fig. 3B,E,H,K). Addition of sulfate rescues the expression of CS and HS chains in chlorate-treated *+/+* and *Lp/+* embryos (Fig. 3C,F,I,L), generating a staining pattern closely resembling that of controls (Fig. 3A,D,G,J). This chlorate-specific reduction in GAG sulfation correlates closely with Closure 1 inhibition in *Lp/+* embryos, although notably the inhibition of CS and HS immunostaining is present also in +/+ embryos, which are much less often affected by chlorate.

### Enzymatic cleavage of GAG chains recapitulates the phenotype of chlorate treatment in *Vangl2^Lp/+^* embryos

**Fig. 4.**
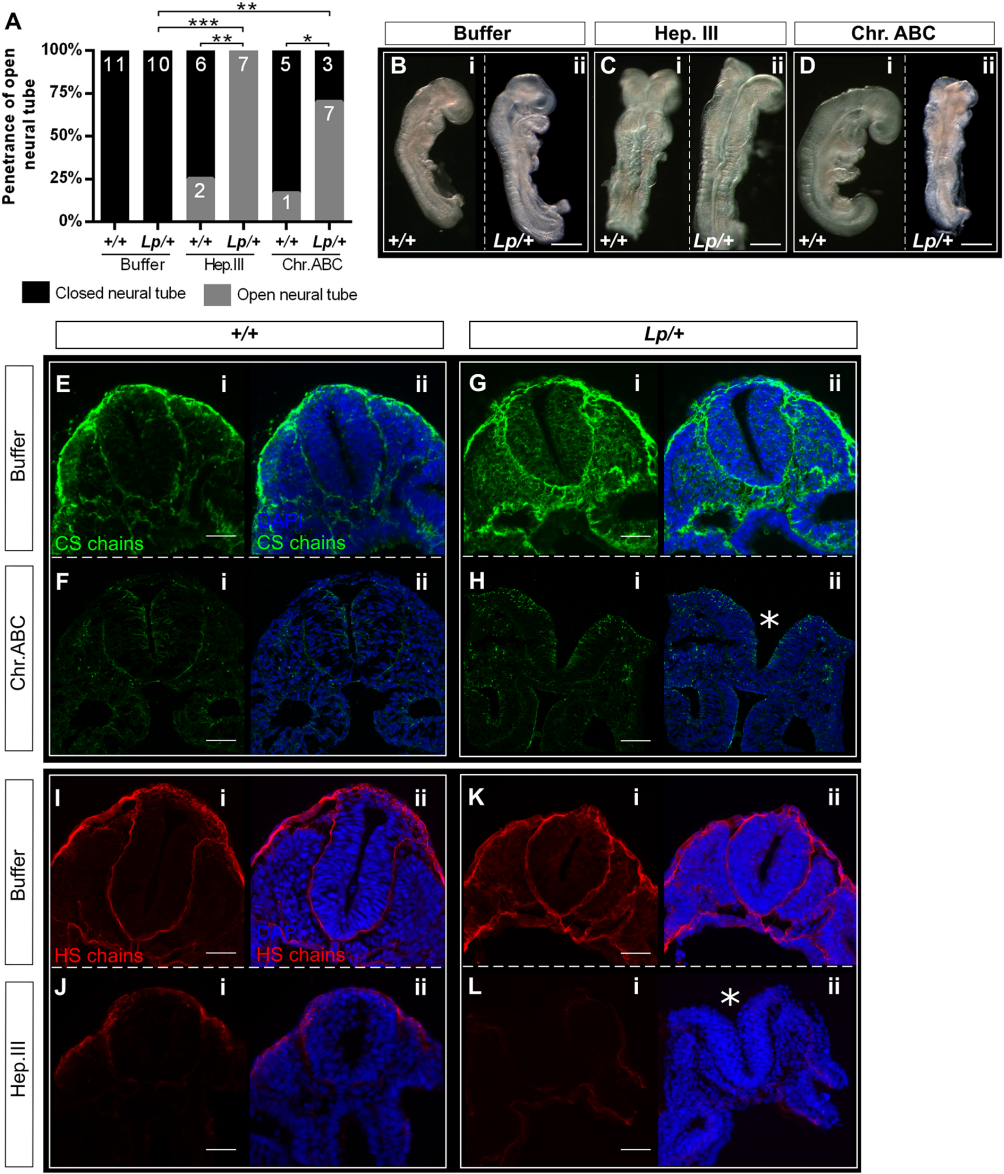
**Enzymatic cleavage of CS and HS chains induces Closure 1 failure in *Lp/+* embryo cultures.** Heparitinase III (Hep.III; to cleave HS chains, 5 U/ml) or chondroitinase ABC (Chr.ABC; to cleave CS chains, 2 U/ml) was injected into the amniotic cavity of E8.5 embryos at 0- to 5-somite stage (prior to Closure 1) at time 0, and injection was repeated after 4 h of culture. Embryos were cultured for 24 h in total. (A) All *+/+* and *Lp/+* embryos from the control group (enzyme buffer only) completed Closure 1. Both Hep.III and Chr.ABC significantly inhibited Closure 1, producing open NT (grey bar sectors) in *Lp/+* embryos, whereas *+/+* embryos remained largely unaffected. Embryo numbers are shown on bars. (B-D) Examples of embryos after culture, showing *+/+* and *Lp/+* buffer-treated embryos with closed NT (B-i,ii), *+/+* embryos with closed NT despite treatment with enzymes (C-i,D-i), and *Lp/+* embryos with failed Closure 1 after Hep.III and Chr.ABC. (E,G,I,K) Both *+/+* and *Lp/+* embryos from the control group (buffer) show the expected distribution of CS/HS chains after culture (compare with Fig. 3A,D,G,J, respectively). (F,H) Chr.ABC injection dramatically reduces the staining of the CS chains in both genotypes. (J,L) *+/+* and *Lp/+* embryos injected with Hep.III display very weak staining of HS chains. Chr.ABC does not affect HS staining, nor does Hep.III affect CS staining (Fig. S4). Asterisks indicate failed neural tube closure in *Lp*/+ embryos treated with Chr.ABC (H-ii) and Hep.III (L-ii). Somite stages: B-i, 12; B-ii, 13; C-i, 14; C-ii, 15; D-i, 14; D-ii, 10; E-G, 12; H, 11; I, 13; J-L, 12. *P<0.05; **P<0.01; ***P<0.001 (Chi-square test). Scale bars: 0.5 mm (B-D); 50 µm (E-L).

Chlorate affects the sulfation of both HS and CS chains (Yip et al., 2002). To address whether one or other type of GAG chain is particularly required for the closure initiation, *+/+* and *Lp/+* embryos with 0-5 somites were cultured following intra-amniotic injection of specific GAG-degrading enzymes: heparitinase III (Hep.III) for HS or chondroitinase ABC (Chr.ABC) for CS. Specificity of these enzymes was demonstrated on embryo sections immunostained for HS or CS, following enzyme treatment in embryo culture (Fig. S4). All *+/+* and *Lp/+* embryos in buffer-treated cultures achieved Closure 1 and exhibited normal morphology (Fig. 4A,B), whereas closure failed, producing an entirely open NT, in all *Lp/+* embryos treated with Hep.III (7/7; *P*<0.001) and in most treated with Chr.ABC (7/10; *P*=0.003) (Fig. 4A,C,D). A small number of *+/+* embryos failed in Closure 1 after treatment with either Hep.III (2/8) or Chr.ABC (1/6), as also observed with chlorate treatment of wild-type embryos (Fig. 2A). The frequency of NT closure failure in enzyme-treated *+/+* embryos did not differ significantly from that in *+/+* buffer-treated controls.

Immunofluorescence analysis of cultured embryos revealed strong immunostaining of CS and HS chains in buffer-treated +/+ and *Lp*/+ embryos (Fig. 4E,G,I,K). In contrast, the staining of CS chains was almost absent in the Chr.ABC treated *+/+* and *Lp/+* embryos (Fig. 4F,H), while exposure to Hep.III dramatically reduced HS staining, with only some SE basement membrane staining persisting in *+/+* embryos (Fig. 4J,L). Hence, enzymatic cleavage of either CS or HS chains recapitulates the Closure 1 phenotype of chlorate-treated *Lp/+* embryos, confirming the requirement for GAGs in early neurulation. Each GAG type appears essential in its own right, perhaps indicating that each performs a distinct function. Alternatively, diminution of either HS or CS might reduce overall sulfated GAG function below a minimum threshold level needed for closure.

### Closure 1 failure in chlorate-treated *Lp/+* embryos is not due to faulty neuroepithelial CE

Mouse embryos homozygous for PCP mutations exhibit abnormal CE cell movements, preceding failure of Closure 1 (Greene et al., 1998; Ybot-Gonzalez et al., 2007; Williams et al., 2014). Moreover, CRN, the severe NTD that results from Closure 1 failure, has been found almost exclusively in association with a short, wide body axis, indicative of defective CE (Curtin et al., 2003; Wang et al., 2006; Yen et al., 2009; Murdoch et al., 2014). We hypothesized that the Closure 1 defects observed in *Lp/+* embryos after chlorate treatment result from enhancement of a heterozygous predisposition to faulty CE. Vital labelling of the embryonic midline at the late-gastrulation stage has shown that *Lp/Lp* mutants fail in CE both in NE and axial mesoderm, prior to the stage of Closure 1 (Ybot-Gonzalez et al., 2007). Here, we used a similar labelling technique to investigate the effect of chlorate on CE in *Lp/+* embryos. *Lp/Lp* mutants served as ‘positive controls’, as they were expected to show reduced CE even under normal culture conditions.

**Fig. 5.**
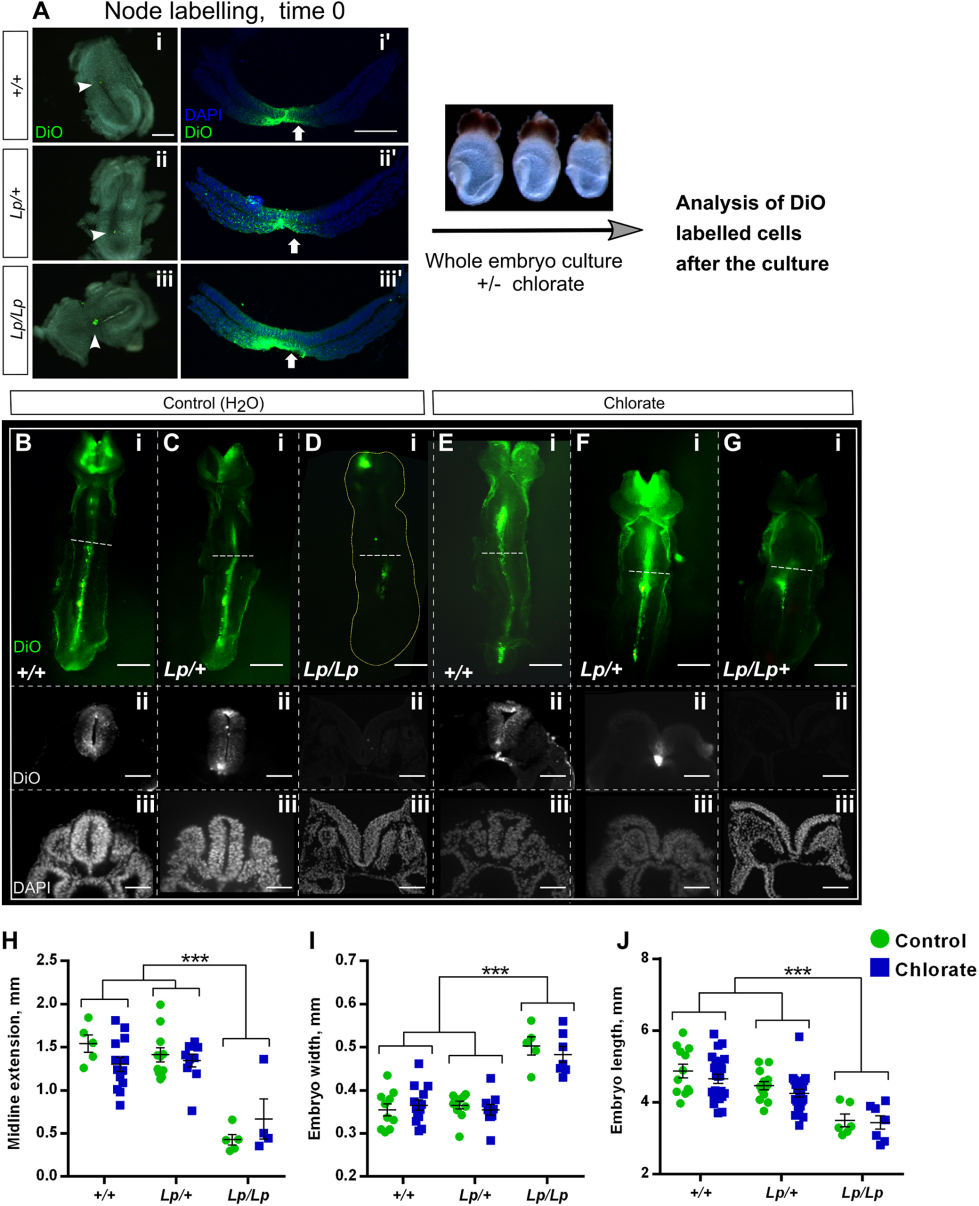
**Chlorate prevents Closure 1 without affecting midline extension of *Lp/+* embryos.** (A) The node of E8.5 embryos from *Vangl2^Lp/+^*×*Vangl2^Lp/+^* matings (0- to 5-somite stage, blind to genotype) was labelled by microinjection of the lipophilic fluorescent dye (DiO) (A-i,ii,iii). Embryos were then randomly allocated to 10 mM chlorate or water treatment and cultured for 24 h. Transverse sections through embryos at time 0 show the node and floor plate are successfully labelled with DiO in all three genotypes (A-i′,ii′,iii′). (B-G) Ventral view (rostral to the top) and transverse sections (trunk region) of DiO-injected embryos, after 24 h culture. Control (water) and chlorate-treated *+/+* and *Lp/+* embryos (B,C,E,F) exhibit marked midline extension of DiO-labelled cells, as detected in both whole mount and sections. Sections reveal failed Closure 1 in chlorate-treated *Lp/+* embryos (F-ii,iii). In contrast, *Lp/Lp* embryos from both treatment groups display very limited midline extension of DiO-labelled cells (D-i,G-i), and fail in Closure 1 (D-ii,iii,G-ii,iii). Note that DiO labelling of the cranial region is non-specific, due to release of DiO into the amniotic cavity during labelling. (H) Midline extension measurements: points represent the distance DiO-labelled cells have extended along the caudal-to-rostral axis in individual embryos, with mean±s.e.m. also shown. *Lp/Lp* embryos exhibit significantly less midline extension than other genotypes. Chlorate-treated *Lp/+* embryos do not differ from *+/+* (water or chlorate) or *Lp/+* (water) groups. (I,J) Embryo width (I) and length (J) measurements reveal a significantly wider and shorter body axis in *Lp/Lp* embryos than in other genotypes, irrespective of water/chlorate treatment. Width and length of *Lp/+* embryos do not differ significantly between water and chlorate groups, nor do these values differ from *+/+* embryo measurements. Data points are individual measurements, with mean±s.e.m. values also shown. *** *P*<0.001 (two-way ANOVA). Scale bars: 50 µm (B-ii,iii,C-ii,iii,D-ii,iii,E-ii,iii,F-ii,iii,G-ii,iii); 100 µm (A-i′,ii′,iii′); 200 µm (A-i,ii,iii); 0.5 mm (B-i,C-i,D-i,E-i,F-i,G-i).

The embryonic midline was labelled by focal injection of DiO into the node at E8.5 in *+/+*, *Lp/+* and *Lp/Lp* embryos, prior to culture with or without chlorate (Fig. 5A). Analysis of transverse embryo sections immediately after labelling (time 0) revealed that both node and floor plate were successfully labelled with DiO in all three genotypes (Fig. 5Ai′,ii′,iii′). Analysis after 24 h culture revealed that both *+/+* and *Lp/+* embryos from the water-treated control group exhibit rostrally directed midline extension of DiO-labelled cells (Fig. 5B,C). Moreover, chlorate-treated *+/+* and *Lp/+* embryos also exhibit rostrally directed midline extension of DiO-labelled cells (Fig. 5E,F), closely resembling the water-treated controls. In contrast, *Lp/Lp* embryos display very limited midline extension, whether treated with water (Fig. 5D) or chlorate, with the latter having no discernible effect on midline extension in *Lp/Lp* embryos (Fig. 5G). Labelled cells were persistently located at the site of node injection in *Lp/Lp* embryos, as described previously (Ybot-Gonzalez et al., 2007). Transverse sections demonstrate that, following 24 h culture, labelled cells can be detected extending along the midline, from the caudal region to upper trunk in *+/+* and *Lp/+* embryos, with no obvious difference between water or chlorate treatments (Fig. 5B-ii,C-ii,E-ii,F-ii). In contrast, midline extension of DiO-labelled cells was not detected in the notochord and floor plate of *Lp/Lp* embryos, either water (Fig. 5D-ii) or chlorate (Fig. 5G-ii) treated.

Midline extension was quantified by measuring from the caudal end of the embryo to the rostral limit of continuous midline DiO signal, which typically ended around heart level (Fig. 5H). Chlorate treatment did not affect midline extension overall (two-way ANOVA, *P*=0.92), and there was no statistically significant interaction between genotype and treatment (*P*=0.21). Midline extension varied significantly between genotypes (*P*<0.001), as a result of the greatly reduced extension in *Lp/Lp* embryos, whereas *+/+* and *Lp/+* genotypes did not differ significantly from each other (*P*=0.41).

We evaluated embryo width and length after culture, as a further measure of CE (Fig. 5I,J). Chlorate treatment did not significantly affect embryo width and length overall (*P*=0.28 and *P*=0.44, respectively) and there was no statistically significant interaction between genotype and treatment (*P*=0.27 for width; *P*=0.89 for length). Embryo width and length varied significantly between genotypes, with both *+/+* and *Lp/+* embryos exhibiting smaller width and increased length compared with *Lp/Lp* littermates (*P*<0.001 for both). Whether treated with chlorate or water, *+/+* and *Lp/+* embryos do not differ from each other in either width or length (*P*>0.05). In conclusion, chlorate treatment does not appear to disrupt the process of neuroepithelial CE in *Lp/+* embryos, even though they fail in Closure 1 and develop CRN at high frequency. As expected, *Lp/Lp* embryos exhibit defective CE, whether treated with water or chlorate.

### Effect of chlorate treatment on neural plate morphology in the Closure 1 region

**Fig. 6.**
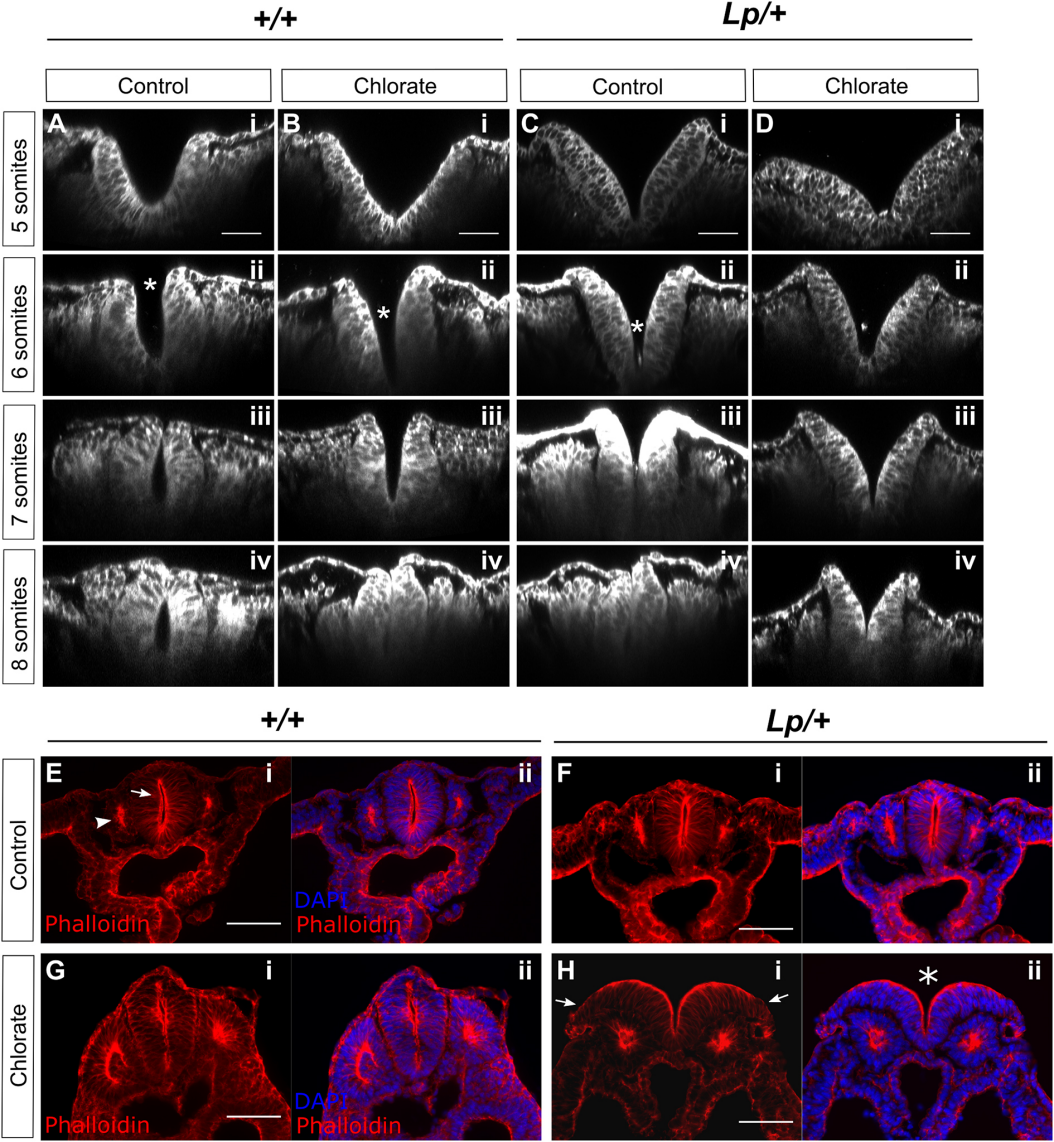
**Chlorate alters neural plate morphology but not overall F-actin distribution in cultured embryos.** (A-D) Embryos were cultured for 8 h with addition of 10 mM chlorate or water as control, fixed, stained with CellMask™ and imaged using confocal microscopy for morphological analysis. Images were re-sliced in Fiji to obtain transverse sections of the Closure 1 region, at the level of the 3rd somite. *+/+* (A,B) and *Lp/+* (C,D) embryos with 5, 6, 7 or 8 somites are shown (i-iv for each genotype/treatment combination). Closure 1 is normally completed from the 6-somite stage onwards. In control (water-treated) *+/+* embryos, the neural plate adopts an increasingly horseshoe shape, concave inwards, with fusion evident dorsally from 7 somites (A). Chlorate delays this transition in *+/+* embryos, with an initially V-shaped morphology, but closure is achieved by 8 somites, when the neural tube appears largely normal (B). Water-treated *Lp/+* embryos resemble chlorate-treated *+/+* embryos, and achieve closure by 8 somites (C). Chlorate-treated *Lp/+* embryos exhibit a persistently V-shaped neural plate with convex curvature, in which the dorsal aspects of the neural folds fail to converge and fusion fails (D). Asterisks in A-ii, B-ii and C-ii indicate sites of initial contact between neural folds. (E-H) Phalloidin staining to detect F-actin distribution in transverse sections of the Closure 1 region of embryos cultured for 24 h. F-actin is enriched at the apical surface of NE (arrow in E-i) and apically within the epithelial somites (arrowhead in E-i). Although failure of NT closure is seen in chlorate-treated *Lp/+* embryos (asterisk in H-ii), the only obvious difference from *+/+* (E,G) and water-treated *Lp/+* embryos (F) is an apparently reduced intensity of phalloidin staining at the lateral edges of the open neural folds (arrows in H-i) (*n*=4 embryos each). Scale bars: 50 µm (A-D); 100 µm (E-H).

To further investigate the embryonic mechanisms leading to failure of NT closure in chlorate-treated *Lp/+* embryos, we examined the morphology of mid-axial tissues during the onset of Closure 1. Cultures were started at the 0- to 4-somite stage (prior to Closure 1) and continued for 8 h (as opposed to 24 h cultures in previous experiments), in the presence of 10 mM chlorate or water as control. Neural plate morphology of the Closure 1 region was then examined in *+/+* and *Lp/+* embryos at the 5-, 6-, 7- and 8-somite stages. Embryos were whole-mount stained with CellMask™ to label all tissues, followed by confocal imaging and ‘re-slicing’ in Fiji to obtain virtual transverse sections of the Closure 1 region, at the level of the 3rd somite (Fig. 6).

Confocal microscopy revealed the typical ‘horseshoe’ morphology of the NE at the Closure 1 site in *+/+* embryos in the control group. All regions of the neural plate appear to bend in embryos with 5-7 somites so that the neural fold tips come together and fuse in the dorsal midline at the 7-somite stage. A completely closed neural tube is present by 8 somites (Fig. 6A). Chlorate treatment affected the neural plate morphology of *+/+* embryos, in which a well-defined bend occurred at the neural plate midline at the 5- and 6-somite stages (Fig. 6B). This resembles the MHP that characterizes closure at upper spinal levels, following completion of Closure 1 (Shum and Copp, 1996). Hence, the horseshoe morphology was less obvious in chlorate-treated embryos. Nevertheless, their neural folds invariably came into contact and appeared to fuse just below the dorsal tips (asterisk in Fig. 6B-ii), thereby completing Closure 1 by the 8-somite stage.

*Lp/+* embryos in the water-treated group also exhibited a ‘V’-shaped neural plate at the 5- and 6-somite stages (Fig. 6C). Their neural folds elevated and came into contact apparently in a ventral-to-dorsal sequence within the NE (Fig. 6C-ii,iii), rather than by initial contact between the neural fold tips (Fig. 6A-ii,iii). Closure 1 was completed by the 8-somite stage. The neural plate of chlorate-treated *Lp/+* embryos had a similar morphology to that of water-treated *Lp/+* embryos at the 5- and 6-somite stage (Fig. 6D), but the distance between neural fold tips was greater, as a result of bilateral convexity of the NE. This was observed at all stages up to and including 8 somites, and hence Closure 1 failed in most of these embryos.

Measurements of the distance between the neural fold tips confirmed that both chlorate-treated *+/+* and water-treated *Lp/+* embryos exhibit delayed Closure 1 compared with normally developing, water-treated *+/+* embryos. Moreover, chlorate-treated *Lp/+* embryos show no reduction in inter-fold distance with increasing somite number and fail in Closure 1 (Fig. S5).

### Chlorate does not perturb the distribution of F-actin in the neural folds

The finding that chlorate-treated *Lp/+* embryos exhibit convex, rather than concave, neural folds suggested a possible mechanism whereby chlorate may disrupt the NE actin cytoskeleton leading to failure of Closure 1. Apically arranged F-actin microfilaments are well known to participate in neural tube closure, at least in part by biomechanically stabilizing the apical NE and promoting concave curvature (Sawyer et al., 2010; Suzuki et al., 2012). To examine this possibility, we performed phalloidin staining to reveal F-actin distribution in the Closure 1 region of embryos cultured for 24 h. Transverse sections demonstrate an F-actin enrichment at the apical surface of the NE in *+/+* and *Lp/+* embryos from the water-treated control group (Fig. 6E,F), with prominent staining also of the central (apical) region of the epithelial somites that flank the closed neural tube. Chlorate-treated *+/+* embryos display a similar F-actin distribution on the apical aspects of the NE and epithelial somites (Fig. 6G). In chlorate-treated *Lp/+* embryos with an open NT, the majority of each neural fold has prominent F-actin localization apically, including in regions of the greatest convex curvature. We conclude that there is no major alteration in F-actin distribution that can account for the failure of Closure 1 in chlorate-treated *Lp/+* embryos, although this analysis does not rule out a mechanism based on defective actomyosin functioning.

### Possible somite-mediated mechanism of Closure 1 failure in chlorate-treated *Lp/+* embryos

**Fig. 7.**
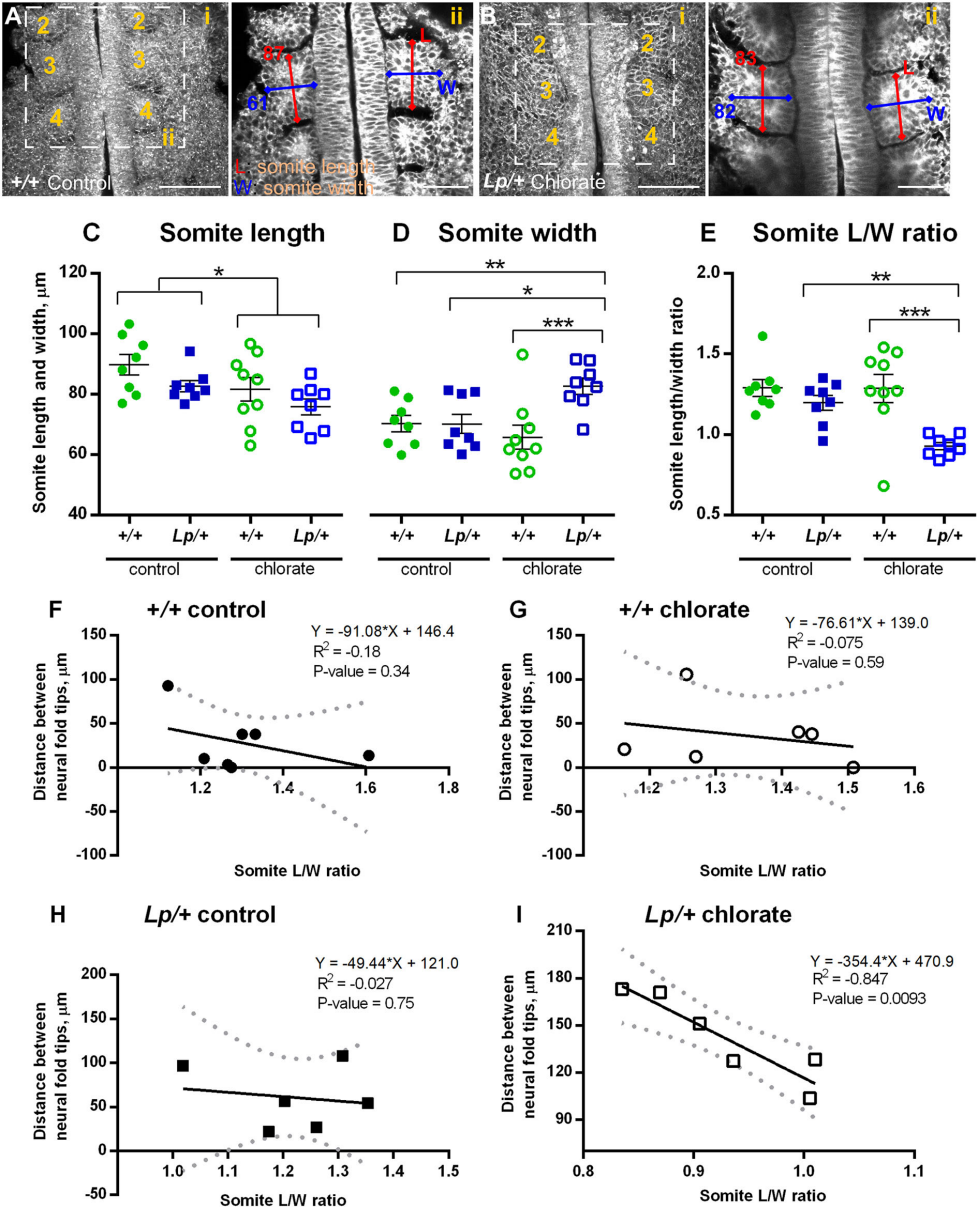
**Altered somite morphology and correlation with Closure 1 delay in *Lp/+* embryos after chlorate treatment.** Embryos were cultured for 8 h from 0- to 5-somite stage, with addition of 10 mM chlorate or water as control, and prepared for morphological analysis as in Fig. 6A-D. (A-i,B-i) *Z*-projections of water-treated *+/+* embryo (A-i) and chlorate-treated *Lp/+* embryo (B-i), both at the 7-somite stage. Rostral is to the top; somites numbered sequentially. Images were re-sliced in Fiji (from dorsal to ventral surface) to obtain horizontal sections through the somite row. (A-ii,B-ii) Single *z*-planes through the Closure 1 region of the same embryos as in A-i and B-i. Length (L; rostrocaudal dimension) and width (W; mediolateral dimension) measurements were taken half way through the somite, at the level of the 3rd and 4th somites. Two somite pairs (4 individual somites) were measured per embryo. The L and W measurements (in µm) of the 3rd somite are shown for both embryos. (C-E) Somite length, width and L/W ratio in control and chlorate-treated *+/+* and *Lp/+* embryos, at the 6- to 7-somite stage. Individual points on the graphs are measurements averaged over 4 somites for each embryo, with mean±s.e.m. of embryo replicates. Note that somite width is significantly increased, and L/W ratio significantly reduced, in chlorate-treated *Lp/+* embryos. *P<0.05; **P<0.01; ***P<0.001 (two-way ANOVA). (F-I) Linear regression analysis of distance between neural folds (µm) at the Closure 1 site and somite L/W ratios, with 95% confidence intervals (dotted lines) and a best fit line (solid lines). No significant correlation is detected for *+/+* embryos, in either control (F) or chlorate (G) groups, nor for water-treated *Lp/+* embryos (H). In contrast, chlorate-treated *Lp/+* embryos (I) show a strong negative correlation (*P*=0.0093), with the greatest closure delay in embryos with the lowest somite L/W ratios (6- to 7-somite stage; *n*=6 in each case).

Closure 1 is unusual in mammalian neurulation as it occurs at a rostrocaudal level where the neural folds are flanked by epithelial somites. Most of spinal closure, and all of cranial closure, occur when the neural tube is adjacent to unsegmented presomitic or cranial mesoderm, respectively. Hence, we investigated whether the somites might play a functional role in promoting Closure 1, with possible defects resulting from chlorate treatment, especially in *Lp/+* embryos. Somite length (rostrocaudal) and width (mediolateral) were measured in confocal images at the level of the 3rd and 4th somites of embryos cultured for 8 h, to the 6- to 7-somite stage, when onset of Closure 1 is underway (Fig. 7A,B). Single *z*-plane measurements showed that the length of somites exceeds their width in water-treated +/+ embryos (Fig. 7A-ii), whereas chlorate-treated *Lp/+* embryos had somites with more equal length and width dimensions (Fig. 7B-ii). Quantitative analysis showed that somite length is significantly reduced by chlorate treatment relative to water-treated control embryos (two-way ANOVA; *P*=0.023; Fig. 7C). Somite length was also reduced in *Lp/+* relative to *+/+* embryos, a difference that just reaches significance (*P*=0.049). The interaction between genotype and treatment was not statistically significant for somite length.

When considering somite width, there is a significant genotype–treatment interaction (*P*=0.015) in which chlorate treatment increased somite width of *Lp/+* embryos relative to chlorate-treated *+/+* embryos and also relative to *Lp/+* embryos from the water-treated control group (Fig. 7D). Somite length/width (L/W) ratio showed a particularly strong genotype–treatment interaction (*P*=0.034), with chlorate-treated *Lp/+* embryos having a significantly reduced L/W ratio compared with the other genotype/treatment groups (*P*=0.017) (Fig. 7E). The L/W ratios of *+/+* embryos and water-treated *Lp/+* embryos are 1.2-1.3, indicating that their somites are elongated rostrocaudally, whereas L/W ratio in chlorate-treated *Lp/+* embryos is close to 1.0, showing these embryos to have somites that are as wide as they are long.

The finding of reduced somite L/W ratio in chlorate-treated *Lp/+* embryos suggested a possible association of somite defects with Closure 1 failure. To explore this further, we quantified the degree of neurulation delay in each embryo, by measuring the distance between left and right neural folds across the midline, and plotted this against the somite L/W ratio for the same embryo. Inter-neural fold distance was not significantly related to somite L/W ratio in *+/+* embryos of either group (Fig. 7F,G) or in water-treated *Lp/+* embryos (Fig. 7H). In contrast, chlorate-treated *Lp/+* embryos showed a strong negative correlation between inter-neural fold distance and somite L/W ratio (Fig. 7I, *R*^2^=0.847, *P*=0.0093). This suggests that, on an individual embryo basis, the degree of abnormality of somite morphology is predictive of the degree to which neural tube closure is delayed in chlorate-treated *Lp/+* embryos.

## DISCUSSION

In the present study, we asked whether interactions between the PCP signalling pathway and molecules of the cell surface and extracellular matrix (ECM) may play a role in the initial event of mouse NT closure. We particularly focused on sulfated GAG chains that decorate proteoglycans. Heterozygosity for the *Vangl2^Lp^* PCP mutant allele is ordinarily compatible with initiation of NT closure, but we find a high frequency of Closure 1 failure when synthesis or presence of sulfated GAG chains is also abolished. Hence, partial loss of PCP function can give rise to a severe NTD when combined with loss of GAG sulfation during early embryogenesis.

In zebrafish and *Xenopus*, the axial tissues including neural plate, notochord and mesoderm undergo PCP-dependent mediolateral convergence and rostrocaudal extension (Tada and Heisenberg, 2012). Moreover, ECM proteins and their receptors play a role in CE, with perturbation of ECM components giving rise to defective CE (Topczewski et al., 2001; Skoglund and Keller, 2010). Indeed, PCP signalling and ECM can integrate at the molecular level to regulate CE during neurulation of lower vertebrates. For example, recruitment of *Dsh* (a key intracellular PCP component) to the cell membrane is dependent on fibronectin–integrin and Sdc4–fibronectin interactions in *Xenopus* CE (Munoz et al., 2006).

There have so far been few studies on PCP signalling in the context of ECM dysfunction in higher vertebrate CE. Our finding of Closure 1 failure in *Lp/+* embryos with under- or non-sulfated GAGs led to an initial hypothesis of defective CE, with PCP heterozygosity summating with ECM disturbance to prevent normal shaping of the body axis. However, our vital labelling experiments in embryo culture refute this hypothesis. *Lp/+* embryos undergo normal midline extension of DiO-labelled node-derived cells, with or without exposure to chlorate. In contrast, PCP-compromised *Lp/Lp* embryos show greatly reduced midline extension, confirming that defective CE is a major defect in the absence of PCP function in mice (Ybot-Gonzalez et al., 2007) as well as in other vertebrates (Wallingford et al., 2002). Furthermore, morphological features of defective CE (increased width and reduced length) are not present in chlorate-treated *Lp/+* embryos. Hence, faulty Closure 1, with subsequent development of CRN, the most severe NTD in mice and humans, is not solely the outcome of defective neuroepithelial CE, but can also arise from another embryonic abnormality in which partial loss of PCP signalling plays a non-exclusive role.

We found that inhibition of GAG sulfation changes the morphology of the Closure 1 region, prior to the normal stage of closure, suggesting that proteoglycans are involved, directly or indirectly, in regulation of neuroepithelial bending. Loss of Sdc4, an HS proteoglycan with a minority of CS chains, interacts with the *Vangl2^Lp^* allele, disrupting neural tube closure in the low spine (Escobedo et al., 2013). However, Closure 1 appears normal in these mice, suggesting that the CS/HS requirement for Closure 1 may involve other proteoglycan(s). Chlorate is expected to abolish sulfation of all proteoglycan species, without altering core protein synthesis, and so we cannot determine from this study which proteoglycans may be specifically required for Closure 1. Moreover, the effect of chlorate on neural plate morphology was observed in both *+/+* and *Lp/+* embryos, arguing for a PCP-independent requirement for GAG sulfation. Chlorate-treated *+/+* individuals lose the normal horseshoe morphology of the NE, and show delayed neural fold apposition and closure, although they are able to complete closure in most cases. By contrast, *Lp/+* embryos display more severe abnormalities of neural plate morphology, with a prominent midline bend (MHP) that is not usually seen at this axial level. This defect is most severe in chlorate-treated *Lp/+* embryos, in which the highest frequency of Closure 1 failure occurs.

Apical constriction of neuroepithelial cells due to cytoskeletal actomyosin contraction is often viewed as the ‘principal motor’ that drives neural plate bending and closure (Sawyer et al., 2010; Suzuki et al., 2012). We recently showed that mediolateral polarization of F-actin is regulated by Vangl2. Conditional deletion of Vangl2 from neural plate and SE prevented elevation of the caudal neural folds and caused spina bifida, although Closure 1 occurred normally (Galea et al., 2018). In the present study, Vangl2 was found to be co-expressed with GAG chains in the neural plate, somitic mesoderm and SE of the Closure 1 region, suggesting several potential sites of Vangl2-proteoglycan interaction. However, no obvious abnormalities were found in the intensity and localization of actin following culture with chlorate. In particular, strong apical actin staining was present at sites of neural plate convexity in chlorate-treated *Lp/+* embryos, suggesting that deficiency of apical cytoskeletal components may not be the primary defect leading to failure of Closure 1. Nevertheless, further studies of actomyosin function would be needed to rule out a cytoskeleton-mediated mechanism.

A few mouse mutants have been described in which Closure 1 failure and CRN are observed, in the absence of overt PCP dysfunction. *Fgfr1* null embryos have severe defects including lack of neural tube closure and absence of somites throughout the body axis (Deng et al., 1994; Yamaguchi et al., 1994). After backcross to the C57BL/6 background, knockouts survive longer but CRN is still observed, as is failed somite formation (Hoch and Soriano, 2006). Nap1 (also known as Nckap1) is a regulatory component of the WAVE complex, which regulates the actin cytoskeleton and couples extracellular signals to polarized cell movement. It is expressed in both mesoderm and neural plate, with *Nap1*-deficient embryos displaying a number of developmental abnormalities including delay of mesoderm migration, absent somitogenesis and failure of Closure 1 (Rakeman and Anderson, 2006). Loss of *Oct4* (also known as *Pou5f1*) gene function after embryonic day (E)7.5 causes CRN, random heart tube orientation, failed turning, defective somitogenesis and posterior truncation (DeVeale et al., 2013). Hence, all of these gene defects cause CRN, and it is striking that in each case severe abnormalities of somite formation accompany failed Closure 1, consistent with a possible causal connection. Nevertheless, these mutants all exhibit severely disrupted development, raising the alternative possibility that Closure 1 failure could be a non-specific morphogenetic disruption.

The presence of epithelial somites directly flanking the closing neural folds is a feature of Closure 1 that is not shared with neurulation at more rostral and caudal levels, where unsegmented mesoderm is adjacent to the closing NT. The level at which NT closure initiates is consistently adjacent to the early somites in mammals, as seen in mouse, human, rabbit and pig embryos (Greene et al., 1998; Peeters et al., 1998; Van Straaten et al., 2000; O'Rahilly and Müller, 2002). In chick, NT closure was found to occur by ‘buttoning’ in register with the somites, suggesting that the somites may be involved in enhancing elevation and apposition of the neural folds (Van Straaten et al., 1996). Indeed, presomitic mesoderm has been shown to compress the neural tube and notochord during axial elongation (Xiong et al., 2020).

In the neurulation-stage mouse embryo, CS proteoglycans are particularly abundant in the epithelial somites, and may play a mechanical role in maintaining their integrity and structure. The carboxyl and sulfate groups of GAGs trap water between their chains, generating a Donnan osmotic equilibrium that is responsible for tissue compressive stiffness, as in cartilage (Chahine et al., 2005). Such a biomechanical mechanism appears to operate during mammalian brain closure, where expansion of the unsegmented cranial mesoderm requires synthesis of a hyaluronan-rich extracellular matrix. This increases intercellular spaces and supports the elevating neural folds at the convex stage of midbrain closure (Solursh and Morriss, 1977).

In the present study, the altered L/W ratio of somites was present only in chlorate-treated *Lp/+* embryos, and not in other genotype–environment combinations. Particularly striking was the strong correlation between degree of somite L/W ratio reduction and degree of closure delay, as indicated by the gap between the neural folds in individual embryos. These findings provide support for a possible biomechanical role of the somites in neural fold apposition during Closure 1. As regards a possible mechanism, the short, wide somites in chlorate-treated *Lp/+* embryos could indicate a role for mesodermal CE. *Lp/Lp* embryos, like other mouse PCP homozygotes, exhibit a short, wide body axis in which the somites have reduced rostrocaudal length and increased mediolateral width. This reflects the occurrence of CE not only in neuroepithelium, but also in mesoderm during body axis shaping, as found in *Xenopus*, zebrafish and mice (Keller, 2002; Sutherland et al., 2020; Williams and Solnica-Krezel, 2020). Hence, chlorate could exacerbate a mild CE defect in somite development due to the *Lp/+* genotype, although such an exacerbation was not observed for neuroepithelial CE.

Alternatively, GAG sulfation could play a different role in somite shaping, for example imparting biomechanical properties to the epithelialized somite, which interacts with a mild *Lp/+* CE defect, to produce the observed change in overall somite shape. The lazy mesoderm (*lzme*) mouse mutant harbours a mutation that targets UDP-glucose dehydrogenase, an enzyme of GAG biosynthesis, with mutant embryos displaying defective mesoderm migration during gastrulation (García-García and Anderson, 2003). Hence, the synthesis of GAGs is clearly crucial for presomitic mesoderm development. We hypothesize, therefore, that a biomechanical relationship exists between the epithelial somites and neural plate that is critical for closure at the Closure 1 site. Testing this idea will require specific interventions to alter GAG presence and/or sulfation in somites, as a test of their role in closure initiation.

In terms of human NTDs, heterozygosity for rare, non-synonymous, deleterious variants of PCP genes, including *Vangl2*, has been identified in a number of genomic studies of NTDs, including CRN (Juriloff and Harris, 2012; Robinson et al., 2012). Building on this, the present work suggests that genes encoding proteoglycan core proteins and GAG biosynthetic enzymes may also represent candidate genes that could contribute to risk of human NTDs. Array-based comparative genomic hybridization of a cohort of 189 Caucasian and Hispanic cases with non-syndromic lumbo-sacral myelomeningocele identified heterozygous deletions of glypican genes *GPC5* and *GPC6* (Bassuk et al., 2013) as a significant risk factor. Similarly, ultra-rare deleterious variants in the extracellular matrix genes FRAS1-related extracellular matrix 2 (*FREM2*) and perlecan (*HSPG2*) were found to be associated with myelomeningocele in a cohort of North American individuals (Au et al., 2021). Interestingly, ingestion of chlorate in drinking water has been significantly associated with risk of spina bifida in Italy (Righi et al., 2012). It will be important in future to determine whether rare variants of PCP genes, and genetic or environmental disturbance of sulfated PGs, are present in the same individuals with NTDs and therefore may contribute to NTD pathogenesis through their developmental interaction.

## MATERIALS AND METHODS

### Mouse strains and genotyping

Mouse experiments were conducted under the auspices of the UK Animals (Scientific Procedures) Act 1986 and the Medical Research Council's Responsibility in the Use of Animals for Medical Research (1993). Inbred BALB/c mice were used for immunofluorescence and *in situ* hybridization. *Vangl2^Lp^* mice were maintained on the CBA/Ca background and time-mated with wild-type mice from within the colony to generate embryos. Genotyping for the *Vangl2^Lp^* allele was as described previously (Copp et al., 1994). *Vangl2^flox/flox^* mice (C57BL/6J background) were a gift from Deborah Henderson (Newcastle University). *Vangl2^flox/flox^* were crossed with β-actin*^Cre/+^* mice (C57BL/6J background), to produce *Vangl2^flox/−^;* β-actin*^Cre/+^*. These were backcrossed to *Vangl2^flox/flox^* to remove β-actin*^Cre/+^*, while maintaining the *Vangl2* null allele. The colony was maintained by *Vangl2^flox/flox^*×*Vangl2^flox/−^* matings, which were also used to generate embryos for experiments.

### Embryo culture

Embryos were explanted at E8.5 into Dulbecco's modified Eagle's medium containing 10% fetal bovine serum. Culture was performed in undiluted rat serum, in a roller incubator maintained at 38°C and gassed with a mixture of 5% CO_2_, 5% O_2_, 90% N_2_, as described (New et al., 1973; Pryor et al., 2012). For chlorate experiments, cultures were stabilized for 1 h, and then sterile aqueous sodium chlorate was added (1% volume addition) to a final concentration of 5-20 mM. The same volume of sterile distilled water was added to control cultures. Rescue experiments involved culturing embryos with 10 mM chlorate together with 10 mM exogenous sodium sulfate. GAG-degrading enzymes Chr.ABC (2 U) and Hep.III (5 U), or enzyme buffer, were administered by injection into the amniotic cavity of E8.5 embryos, using a hand-held, mouth-controlled glass micropipette (∼0.2 µl injected per embryo). Embryos were injected before the start of the culture and again after 8 h of culture. Cultures were terminated after 8 or 24 h, inspected immediately for yolk sac shape and circulation (Table S1), and presence or absence of heart beat, and then yolk sacs were removed and stored for genotyping. Embryos were scored for presence or absence of Closure 1, and somites were counted. Embryos were washed in PBS and fixed overnight in ice-cold 4% paraformaldehyde in PBS.

### Whole-mount *in situ* hybridization

*In situ* hybridization was performed as described previously (Mole et al., 2020). Primer sequences for the *Vangl2* RNA probe were 5′-GGATGCTGCTGAAAGGGAGT-3′ (forward) and 5′-GCACCGGATAGTTGGAAGGT-3′ (reverse). Sense and antisense RNA probes were transcribed from linearized plasmid DNA using a DIG RNA Labeling Kit (Roche). Sections were prepared by embedding hybridized embryos in gelatine-albumin and sectioning on a vibratome (Leica VT1000S) at 40 µm thickness.

### Section immunofluorescence

After fixation, embryos were immersed in 20% sucrose solution and placed on ice for at least 1 h. The sucrose solution was replaced with 7.5% gelatine (37°C for 30 min), and embryos were embedded in a block of gelatine after solidification. Blocks were snap frozen in −70°C isopentane and stored at −80°C. Blocks were sectioned on a Leica cryostat at 10 μm thickness and mounted on Superfrost Plus slides (Thermo Fisher Scientific). After defrosting, slides were incubated with 200 µl blocking buffer (10% sheep serum, 0.1% Tween 20 in PBS) for 1 h at room temperature. For immunohistochemistry of Vangl2, blocking was in 5% normal goat serum, 0.1% Triton X-100, 2% bovine serum albumin in PBS. The blocking solution was replaced with primary antibody (Table S2) diluted in blocking buffer, and slides were kept overnight at 4°C (200 µl per slide, covered by parafilm). The following day, slides were washed three times in PBS and incubated in secondary antibody at 1:500 dilution in blocking buffer for 1 h at room temperature. Slides were washed three times in PBS and incubated in 4′,6-diamidino-2-phenylindole (DAPI; 1:10,000) for 3 min, washed three times with PBS and mounted in Mowiol. For F-actin staining, sections and whole embryos [paraformaldehyde (PFA) fixed only] were incubated with phalloidin (1:200; Alexa-Fluor-568–phalloidin, A12380, Life Technologies).

Chr.ABC and Hep.III specificity was confirmed on embryo cryosections, prior to enzyme usage in embryo cultures (Fig. S4). Slides were defrosted, incubated in PBS at 37°C, washed in PBS at room temperature, then incubated with Chr.ABC or Hep.III at 37°C for 1 h (200 µl per slide). Control slides were incubated with the corresponding enzyme buffer. Slides were washed three times with PBS at room temperature, blocked with blocking buffer and then stained with corresponding primary antibodies as above.

### Whole-mount immunofluorescence

After dissection, embryos were rinsed in PBS and fixed in ice-cold 4% paraformaldehyde for 1 h. Embryos were permeabilized using 0.1% Triton X-100 in PBS (PBT solution), then washed for 30 min at room temperature and incubated with blocking solution (5% bovine serum albumin in PBT) overnight at 4°C while rocking. Embryos were incubated with primary antibody overnight at 4°C with rocking. Primary and secondary antibodies were diluted in blocking solution to working concentrations (see Table S2). The next day, embryos were washed three times in blocking solution for 1 h, rocking at room temperature, and incubated with secondary antibody for 2 h under the same conditions. Embryos were washed in blocking solution three times for 1 h, rocking at room temperature, then incubated with DAPI (1:15,000), overnight rocking at 4°C. After washing three times in PBS for 30 min at room temperature, embryos were stored in 0.1% sodium azide in PBS at 4°C, prior to imaging. Confocal images were obtained on a Zeiss LSM880 Observer microscope as described previously (Galea et al., 2017). In some cases (e.g. Vangl2 and E-cadherin double immunostaining; Fig. 1F), the SE and apical surface of the neural plate were ‘isolated’ virtually using an in-house Fiji macro (IdentifyUpperSurfacev2.ijm) (Galea et al., 2018), developed to identify and extract the surface of 3D structures, and available from https://github.com/DaleMoulding/Fiji-Macros.

### Assessment of axial extension by DiO injection into the embryonic node

*Vangl2^Lp/+^×Vangl2^Lp/+^* matings were performed to generate litters containing *+/+*, *Lp/+* and *Lp/Lp* embryos. DiO labelling of the node was performed as described previously (Ybot-Gonzalez et al., 2007; Pryor et al., 2012). Some injected embryos (*n*=2 per treatment/genotype group) were collected at time 0 to verify the injection site, while others were randomly distributed to either 10 mM chlorate or water control groups, and cultured for 20-24 h. After culture, embryos were assessed for health and developmental parameters, yolk sacs were stored for genotyping, and fluorescence images were obtained to determine the degree of axial extension of DiO-labelled cells. Embryos were gently flattened using forceps, with ventral surface upwards, and photographed on the stage of a Leica fluorescence stereomicroscope. To confirm the presence of the dye in the midline, three cultured embryos from each treatment group were cryo-embedded for transverse sectioning. Analysis was performed blind to embryo genotype and treatment group. Distance of DiO midline extension was measured in a rostral direction from the node injection site (in the caudal region). DiO extension occurred typically as far rostrally as heart level. Embryo width was the distance between lateral edges of the somite rows, measured at the level of the 3rd/4th somites, in dorsal view. Embryo length was measured along the dorsal surface of the embryo, from forebrain to tailbud. All measurements were made in Fiji. The presence of DiO in head folds of some embryos is non-specific (caused by release of DiO into the amniotic cavity during node injection).

### Analysis of neural plate and somite morphology

Embryos were fixed in 4% PFA and exposed to CellMask™, which stains membranes non-specifically, then imaged by epifluorescence on an inverted LSM710 or LSM880 with Airyscan confocal system (Zeiss). For neural plate morphology, images were re-sliced in Fiji to obtain transverse sections of the Closure 1 region, located at the level of the 3rd somite. The distance between neural folds was measured at ten sequential positions, 20 µm apart, moving rostrocaudally along the Closure 1 region (200 µm in total). For somite morphology, the images were re-sliced in Fiji (from dorsal to ventral surface) to obtain longitudinal sections through the somites. Length (rostrocaudal orientation) and width (mediolateral orientation) measurements were taken half way through the somite at the level of the 3rd/4th somite. Two somite pairs (4 individual somites) were measured per embryo.

### Statistical analysis

Frequencies of Closure 1 failure in cultured embryos were compared by Chi-square or Fisher's exact test. Yolk sac circulation scores were compared by Kruskal–Wallis one-way ANOVA. Embryo length, width and midline DiO extension were compared by two-way ANOVA (SPSS v.24). Analysis of neural plate morphology was performed in SPSS by mixed model analysis of distance between neural fold tips along the Closure 1 region, with four fixed effects: genotype, treatment, position and somite stage. The first-order interactions between fixed effects were computed per parameter. When the fixed effects were significant overall, a post-hoc Bonferroni correction was used to identify the individual sites at which the effect was significant (*P*=0.05).

## Supplementary Material

Supplementary information
