# Supplementary material for: Vangl2–environment interaction causes severe neural tube defects, without abnormal neuroepithelial convergent extension
Source: Dis Model Mech. 2022 Jan 26;15(1):dmm049194. doi: 10.1242/dmm.049194 (PMC8807581; doi:10.1242/dmm.049194)
Supplement: Supplementary information [file dmm-15-049194-s1.pdf]

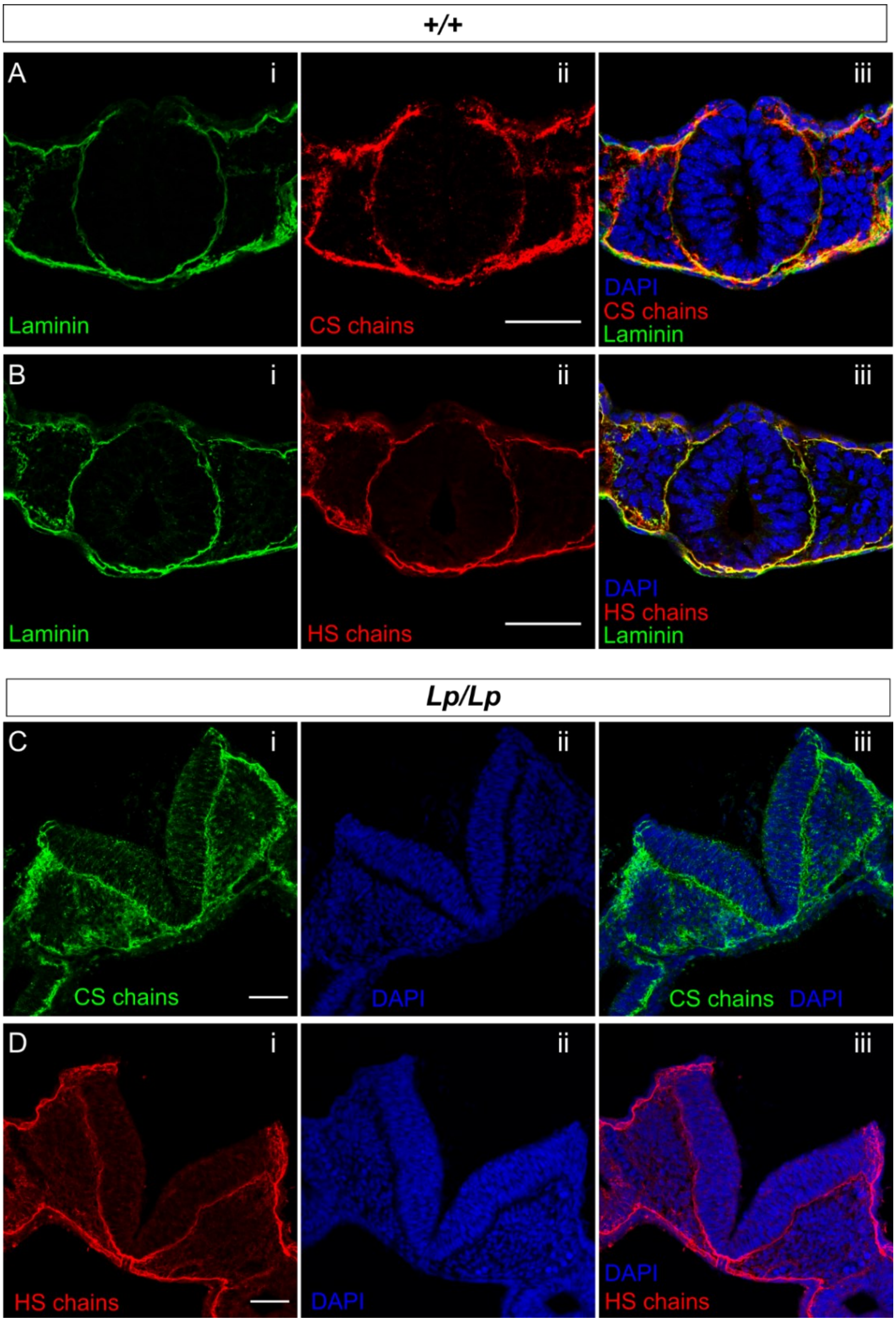

Fig. S1. Co-localization of HS and CS chains with laminin. Immunofluorescence staining on cryosections of +/+ (A,B) and *Lp/Lp* (C,D) embryos. **(A,B)** Double immunohistochemistry: anti-laminin together with anti-CS (A, 6 somite stage) or anti-HS (B, 7 somite stage) antibodies. Laminin co-localizes with CS and HS chains at the BM of neural plate and SE at the Closure 1 site. HS staining shows an almost complete overlap with laminin whereas CS chains are also detected outside the laminin-expressing domain. (**C,D**) Both CS and HS chains localise to the basement membrane of NE and SE in the Closure 1 region of *Lp/Lp* embryos (7 somite stage), with CS chains also present in the somitic mesoderm. Distribution of GAG chains is not markedly different from +/+ embryos, despite failed neural tube closure. Scale bars: 50 µm.

Disease Models & Mechanisms • Supplementary information

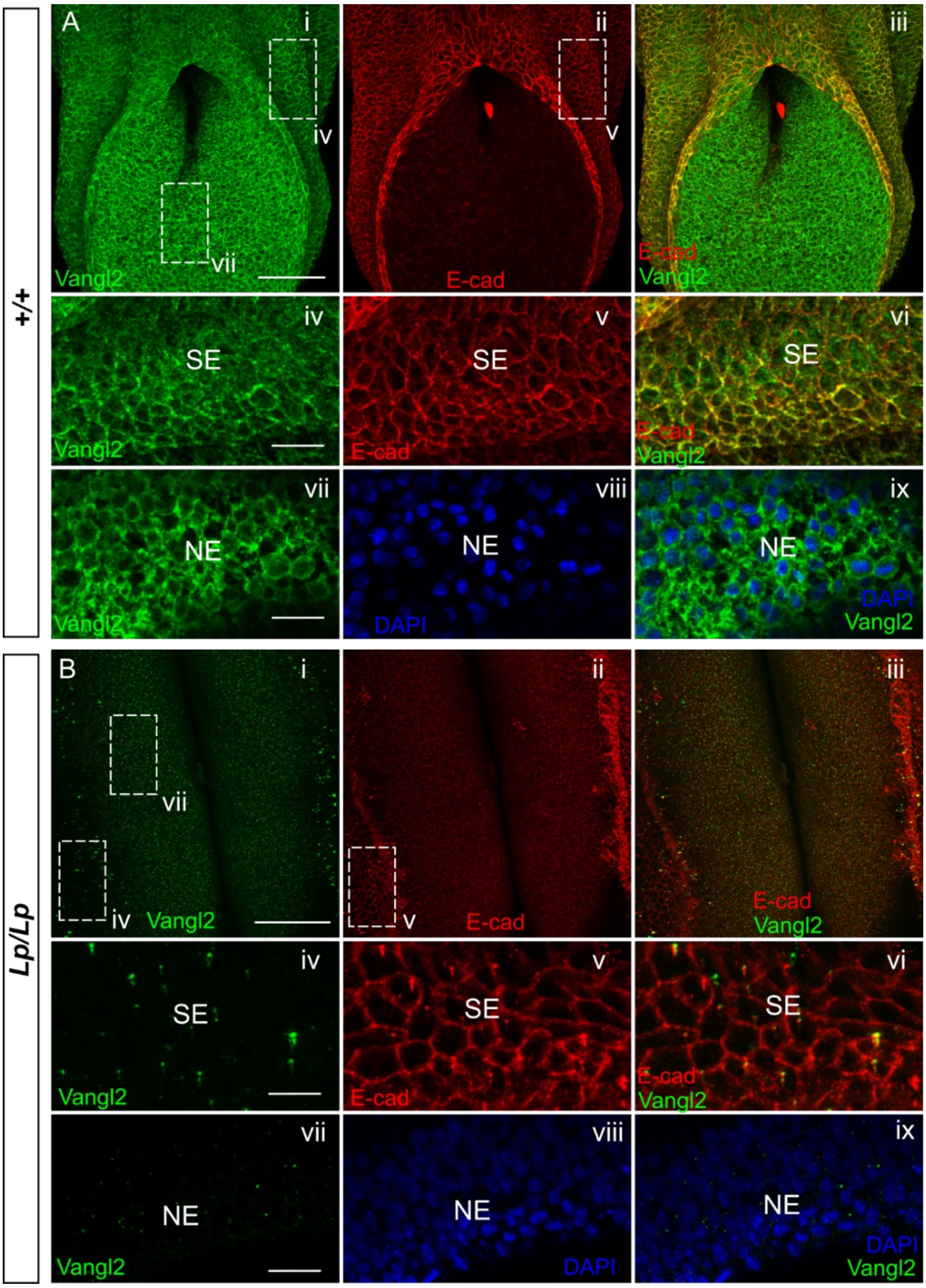

Fig. S2. Comparison of Vangl2 protein in wild-type and *Lp/Lp* mutant embryos.
(A) Dorsal view of the PNP region of a whole mount +/+ embryo (14 somite stage) double immunostained for Vangl2 and E-cadherin (A-i to vi), confirming the presence of Vangl2 in SE at the posterior region. Vangl2 is expressed in broadly expressed in the neuroepithelium (A-vii to ix). **(B)** Dorsal view of caudal region of a whole mount *Lp/Lp* mutant embryo (11 somite stage) double immunostained for Vangl2 and E-cadherin. Vangl2 is absent from the mutant SE (B-i to vi), which expresses only E-cadherin. The mutant neuroepithelium is also negative for Vangl2 (B-vii to ix)**.** Images processed by z-projection (A, B-i to vi) and single z-plane (A, B-vii to ix). Scale bars: A, B-i, ii, iii = 100 µm; A, B-iv to ix = 20 µm.

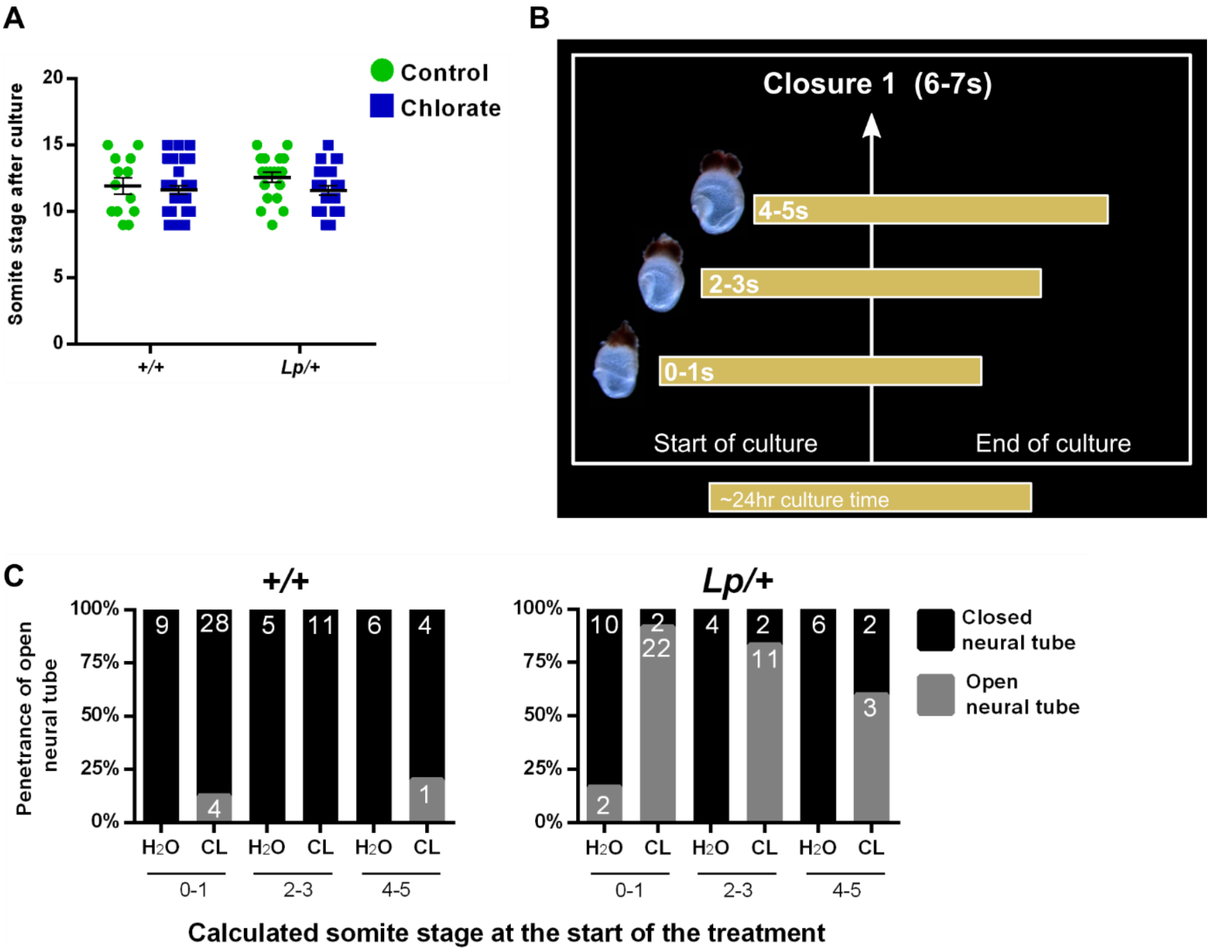

Fig. S3. Relationship between somite stage at start of treatment and Closure 1.
(**A**) Embryo somite stage in +/+ and *Lp/+* embryos after 24 h culture with addition of either water (control) or 10 mM chlorate. Genotypes and treatments do not differ statistically in final somite stage (p = 0.448, p = 0.135 respectively). (**B**) Schematic showing somite stage(s) during the chlorate treatment period in culture, and the timing of Closure 1. Starting somite stage was calculated by subtracting the expected number of somites formed during the culture (2 h = 1 somite; 24 h = 12 somites) from the number of somites recorded at the end of the treatment. One somite was subtracted from the total somite number across all groups, to allow for the time taken to adapt to development in culture. Embryos were pooled into three ‘starting' somite groups: 0-1, 2-3, 4-5. (**C**) Percentage of open/closed +/+ and *Lp/+* embryos, for the calculated somite stages at the start of culture, after exposure to either water (H_2O) or 10 mM chlorate (CL). The number of replicate embryos is indicated in each bar. Most embryos had 0-1 or 2-3 somites at the start of culture (respectively: 61% and 24% for +/+; 56% and 27% for *Lp/+*) and, for these pooled somite ranges, Closure 1 failed in more than 85% of chlorate-treated *Lp/+* embryos. Overall, 17% of embryos started culture in the 4-5 somite range, when it appears that chlorate-treated *Lp/+* embryos could have a lower frequency of Closure 1 failure (3/5; 60%), although numbers are too small for statistical analysis.

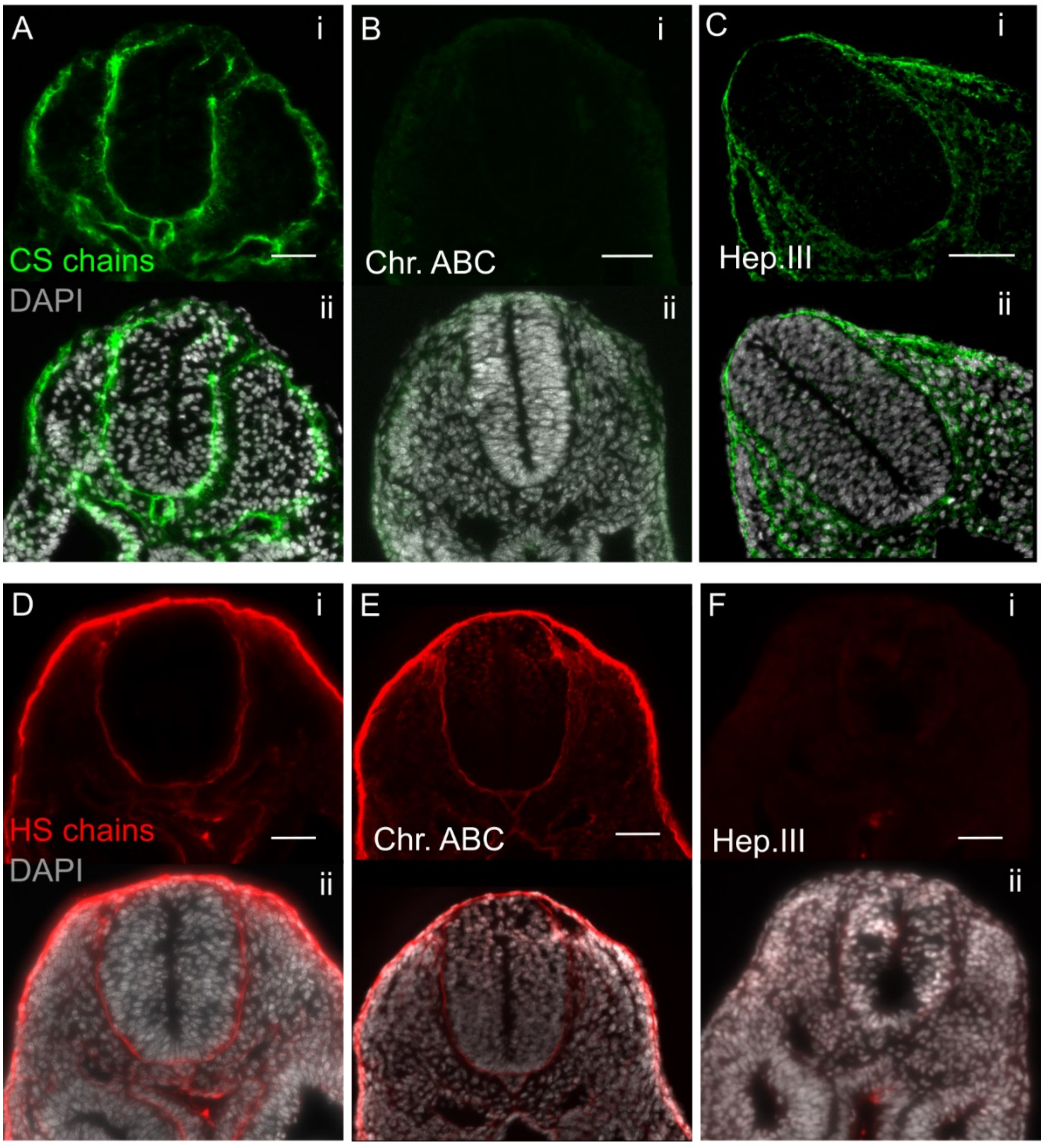

Fig. S4. Validation of GAG proteolytic enzymes and primary antibodies against CS and HS chains. Transverse cryosections through the trunk region of wild type embryos (14- 16 somite stage), pre-incubated with enzyme buffer (A,D), chondroitinase (Chr.) ABC (B,E) or heparitinase (Hep.) III (C,F), prior to immunohistochemical staining for CS or HS chains. Immunofluorescence only (i) and immunofluorescence plus DAPI images (ii) are shown. (**A-C**) CS chains show the distinct basement membrane pattern of staining in buffer-treated controls (A) whereas this is abolished by Chr.ABC pre-treatment (B). Hep.III pre-treatment has minimal effect on CS staining (C). (**D-F**) HS chains are present in basement membranes of SE (strong) and neuroepithelium (weaker) after buffer pre-treatment (D). Hep.III-treated sections show strong reduction of HS staining (F), whereas Chr.ABC does not affect the expression pattern of HS chains (E). Scale bars: 50 µm.

Disease Models & Mechanisms • Supplementary information

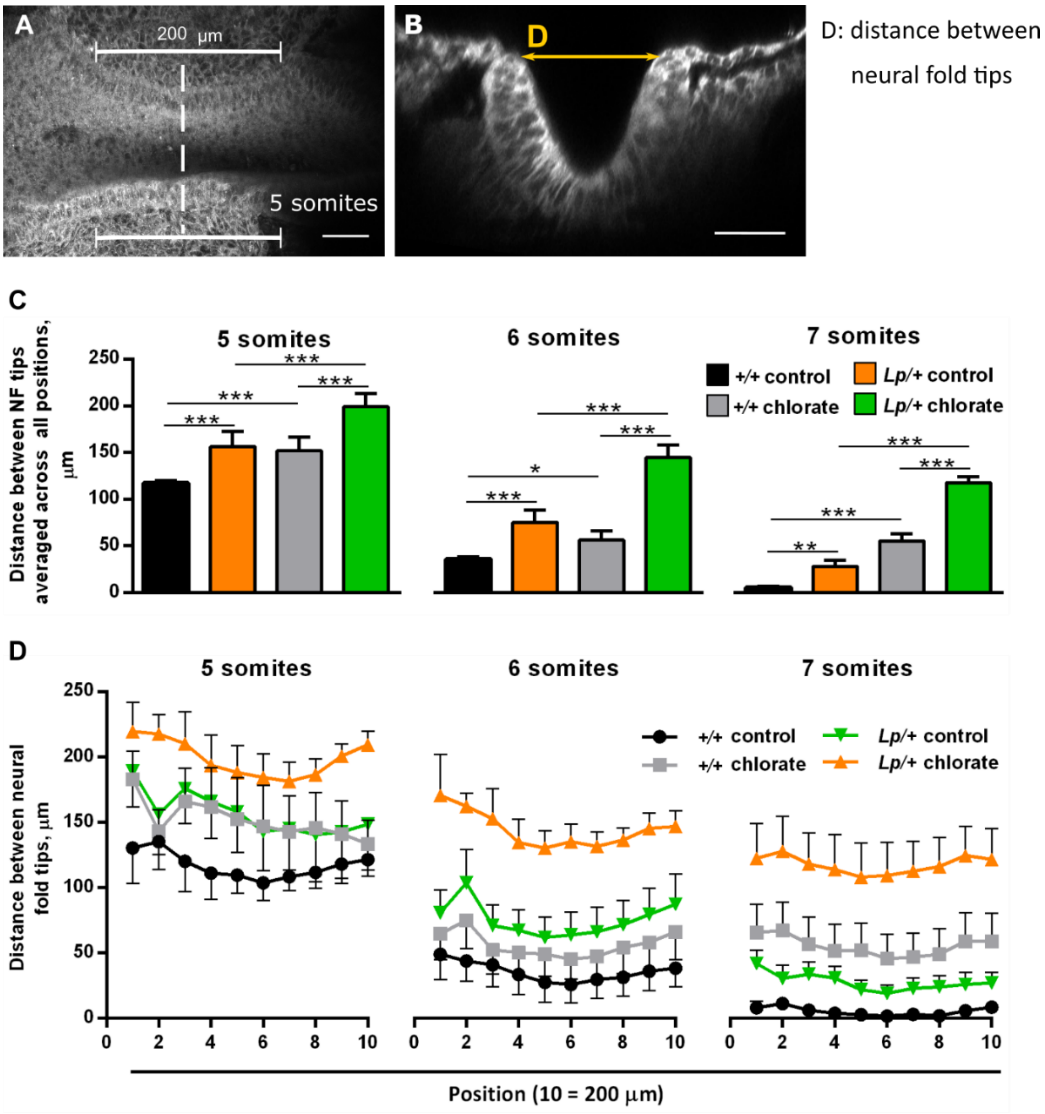

Fig. S5. Quantification of chlorate effects on neural plate morphology at the Closure 1 site. +/+ and *Lp*/+ embryos were cultured for 8 h with addition of 10 mM chlorate or water, fixed, stained with CellMask™ and imaged using confocal microscopy for morphological analysis. **(A)** Dorsal confocal view (rostral to left) showing the converging neural folds at the Closure 1 site of a normally developing embryo. **(B)** Images were re-sliced in Fiji to obtain transverse sections of the Closure 1 region, located at the level of the third somite (along dashed line in A). The distance between the neural fold tips ('D' in B) was measured at 10 sequential positions with 20 µm spacing, moving rostrocaudally along the body axis and centred upon the Closure 1 region. **(C,D)** Distance between neural fold tips averaged across all positions (C) and at individual positions (D) at the 5, 6 and 7 somite stages. Distance between neural fold tips decreases most rapidly with increasing somite stage in +/+ control embryos (black bars, lines), with closure essentially complete at 7 somites. Chlorate-treated +/+ (grey bars, lines) and water-treated *Lp*/+ (green bars, lines) embryos show progressive but delayed closure. Chlorate-treated *Lp*/+ embryos (orange bars, lines) show little progression towards closure. Data values: mean +/- SEM, with a minimum of 4 embryos per genotype/treatment group. * $p < 0.05$; ** $p < 0.01$; *** $p < 0.001$. Scale bar: A,B = 50 µm.

Table S1. Effect of increasing chlorate concentration on embryo health parameters during culture *.

Chlorate conc. (mM)	No. embryos	Health parameters		
		YS circulation score (mean ± SEM)	No. with round YS shape (%)	No. with beating heart (%)
0	21	2.80 ± 0.16	21 (100)	20 (95)
1	12	2.58 ± 0.26	12 (100)	12 (100)
5	32	2.69 ± 0.14	31 (97)	31 (97)
10	41	2.46 ± 0.14	40 (98)	38 (93)
20	21	1.40 ± 0.25	20 (95)	21 (100)

* E8.5 mouse embryos (0-5 somite stage) were cultured for 24 h in the presence of 0, 1, 5, 10 or 20 mM chlorate. Embryo health parameters were measured after the culture by applying scores for: YS (yolk sac) circulation (3-very strong, 2-strong, 1-present, 0- absent); YS shape (1-round, 0-wrinkled); beating heart (1-present, 0-absent). Statistical analysis: Mean YS circulation score varies significantly between groups (p < 0.001; Kruskal-Wallis 1-way ANOVA), with a significantly lower value for 20 mM chlorate compared with all other groups, which do not differ from each other.

Table S2. Primary antibodies used in the study.

Target	Supplier, catalogue number	Source, dilution
Chondroitin sulfate	Sigma, C8035	Mouse CS-56, monoclonal IgM, 1:100
Heparan sulfate	US Biological, H1890	Mouse 10E4, monoclonal IgM, 1:100
Laminin	Abcam, ab11575	Rabbit polyclonal, 1:200
Vangl2	Millipore, MABN750	Rat monoclonal, 1:100
E-cadherin	Cell signalling, 4A2	Rabbit IgG, 24E10, 1:200

Disease Models & Mechanisms • Supplementary information
